# Inside and Beyond Color: Comparative Overview of Functional Quality of Tomato and Watermelon Fruits

**DOI:** 10.3389/fpls.2019.00769

**Published:** 2019-06-13

**Authors:** Riadh Ilahy, Imen Tlili, Mohammed Wasim Siddiqui, Chafik Hdider, Marcello Salvatore Lenucci

**Affiliations:** ^1^Laboratory of Horticulture, National Agricultural Research Institute of Tunisia (INRAT), University of Carthage, Tunis, Tunisia; ^2^Department of Food Science and Postharvest Technology, Bihar Agricultural University, Bhagalpur, India; ^3^Dipartimento di Scienze e Tecnologie Biologiche ed Ambientali, Università del Salento (DiSTeBA), Lecce, Italy

**Keywords:** antioxidants, aromas, biosynthetic pathways, carotenoids, *Citrullus lanatus*, *Solanum lycopersicum*, phenolics, vitamin C

## Abstract

The quali-quantitative evaluation and the improvement of the levels of plant bioactive secondary metabolites are increasingly gaining consideration by growers, breeders and processors, particularly in those fruits and vegetables that, due to their supposed health promoting properties, are considered “functional.” Worldwide, tomato and watermelon are among the main grown and consumed crops and represent important sources not only of dietary lycopene but also of other health beneficial bioactives. Tomato and watermelon synthesize and store lycopene as their major ripe fruit carotenoid responsible of their typical red color at full maturity. It is also the precursor of some characteristic aroma volatiles in both fruits playing, thus, an important visual and olfactory impact in consumer choice. While sharing the same main pigment, tomato and watermelon fruits show substantial biochemical and physiological differences during ripening. Tomato is climacteric while watermelon is non-climacteric; unripe tomato fruit is green, mainly contributed by chlorophylls and xanthophylls, while young watermelon fruit mesocarp is white and contains only traces of carotenoids. Various studies comparatively evaluated *in vivo* pigment development in ripening tomato and watermelon fruits. However, in most cases, other classes of compounds have not been considered. We believe this knowledge is fundamental for targeted breeding aimed at improving the functional quality of elite cultivars. Hence, in this paper, we critically review the recent understanding underlying the biosynthesis, accumulation and regulation of different bioactive compounds (carotenoids, phenolics, aroma volatiles, and vitamin C) during tomato and watermelon fruit ripening. We also highlight some concerns about possible harmful effects of excessive uptake of bioactive compound on human health. We found that a complex interweaving of anabolic, catabolic and recycling reactions, finely regulated at multiple levels and with temporal and spatial precision, ensures a certain homeostasis in the concentrations of carotenoids, phenolics, aroma volatiles and Vitamin C within the fruit tissues. Nevertheless, several exogenous factors including light and temperature conditions, pathogen attack, as well as pre- and post-harvest manipulations can drive their amounts far away from homeostasis. These adaptive responses allow crops to better cope with abiotic and biotic stresses but may severely affect the supposed functional quality of fruits.

## Introduction

Tomato (*Solanum lycopersicum* L.) and watermelon [*Citrullus lanatus* (Thunb.) Matsum. & Nakai var. *lanatus*] fruits enter frequently in our diet as fresh or processed products contributing to the intake of antioxidants and high nutritional value bioactives. Although botanically distant, both species show strong similarities in the chemical profiles of some secondary metabolites, especially the carotenoid pigments of ripe fruits. Among these, lycopene represents the compound to which both fruits owe the health-promoting popularity; nevertheless, many other molecules may synergistically contribute to their functional quality and antioxidant properties. With a global production of 183 and 119 million tons, tomato and watermelon fruits are among the main vegetable crops grown and consumed all over the world, and constitute the main sources of dietary lycopene in eastern and western cultures. Their importance on a global level greatly exceeds that of other lycopene containing fruits characterized by lower production (13 and 9 million tons for grapefruit and papaya, respectively) and per capita consumption (FAO STAT, 2019^1^).

Besides, tomato fruit color mutations have phenotypic equivalents in watermelon, suggestive of a similar regulation of pigment and related metabolites’ biosynthesis and accumulation. Actually, several endogenous and exogenous factors affect the amounts and profiles of all these compounds and cross-talk to finely regulate their metabolic pathways, though they are, in turn, influenced by the different ripening physiology and developmental programs of the two fruits.

Although a wide scientific literature is available on the functional properties and secondary metabolism regulation of tomato and watermelon fruits, only recently some studies comparatively evaluated the development of carotenoid pigmentation during ripening (Lewinsohn et al., 2005b; Tadmor et al., 2005) or the alterations of carotenoid profiles during processing (Aguiló-Aguayo et al., 2009; Aganovic et al., 2017). However, in most cases, the other classes of bioactives received little or no consideration. Here, a comparative overview of the recent finding on the biosynthesis, accumulation and regulation of carotenoids, phenolics, aromas and vitamin C is given in order to emphasize the main similarities/differences between the two fruits at full maturity and during ripening and to highlight the rising concerns about the possible harmful effects of excessive bioactive assumption on health.

## Carotenoid in Tomato and Watermelon Fruits

### Chemical Features and Functions

Carotenoids are natural tetraterpenoid pigments largely produced by plants, algae, phototropic bacteria and some mycetes (Enfissi et al., 2017). In green plant tissues, their biosynthesis takes place within chloroplasts, mostly in the inner envelope and the thylakoid bilayer. Chloroplasts accumulate high levels of lutein, β-carotene, violaxanthin, and neoxanthin, but the green of chlorophylls masks their characteristic yellow/orange color (Sun et al., 2018). Within chloroplasts, carotenoids play essential roles as accessory pigments to maximize light harvesting efficiency of photosystems, and act as chemical buffers against photo-oxidation of the cell constituents detoxifying the triplet state chlorophyll molecules and the highly reactive oxygen species (ROS) produced by photosynthesis (Hashimoto et al., 2016).

In Angiosperms, carotenoids are also responsible for the bright yellow to red pigmentation of many organs, especially flowers and fruits, and are the precursors for the synthesis of scents and flavors involved in the attraction of pollinators and seed dispersors. Non-photosynthetic tissues may accumulate carotenoids within chromoplasts, organelles specialized in their massive biosynthesis and sequestration (Sun et al., 2018). Moreover, carotenoids are intermediates of the synthesis of abscisic acid (ABA) and strigolactones (SLs), key phytohormones regulating plant development and environmental stress responses (Al-Babili and Bouwmeester, 2015; Hou et al., 2016).

Besides their central functions in plants, carotenoids are also essential for human nutrition and health. They provide dietary sources of provitamin A and reduce the incidence of some chronic and age-related pathologies, acting as antioxidants and/or via other, yet not fully understood, molecular mechanisms. These embrace the modulation of gene expression, the regulation of cell metabolism and hormone production, as well as the promotion of immune responses and cell gap-junctional communication (Perveen et al., 2015). Carotenoid content and profiles have become, hence, actual quality traits for many horticultural species, directly influencing crop productivity, industrial demand, consumer appeal, nutritional quality and health promoting properties (Yuan et al., 2015).

The intense research resulting from the multifaceted interest on these pigments triggered the identification and characterization of all genes and enzymes involved in carotenoid biosynthetic and catabolic core reactions (Nisar et al., 2015). It is now well established that the enzymes involved in carotenoid metabolism are encoded by nuclear genes, synthesized in the cytosol, translocated within plastids, and sorted to specific organelle sub-domains depending on plastid type and morphology (Sun et al., 2018). In chloroplasts, sub-plastidial proteomic studies located most carotenogenic enzymes in the envelopes, except for violaxanthin de-epoxidase (VDE), which was associated to thylakoids, and zeaxanthin epoxidase (ZEP), found in both membrane systems (Ytterberg et al., 2006; Joyard et al., 2009). Carotenoid metabolites and enzymes were also detected in thylakoid-associated plastoglobules (PGs), lipoprotein particles identified as a site of carotenoid breakdown for apocarotenoid production and trafficking (Rottet et al., 2016).

Regarding chromoplasts, the information on enzyme sub-organelle location is much more fragmented; however, ζ-carotene desaturase (ZDS), lycopene β-cyclase (LYCB), and two β-carotene hydroxylases (BCH1/2) were identified in PGs isolated from red-bell peppers chromoplasts, suggesting a specific function in carotenoid biosynthesis, beyond the known role as storage/sequestering compartments (Siddique et al., 2006).

### Biosynthesis

**FIGURE 1:**
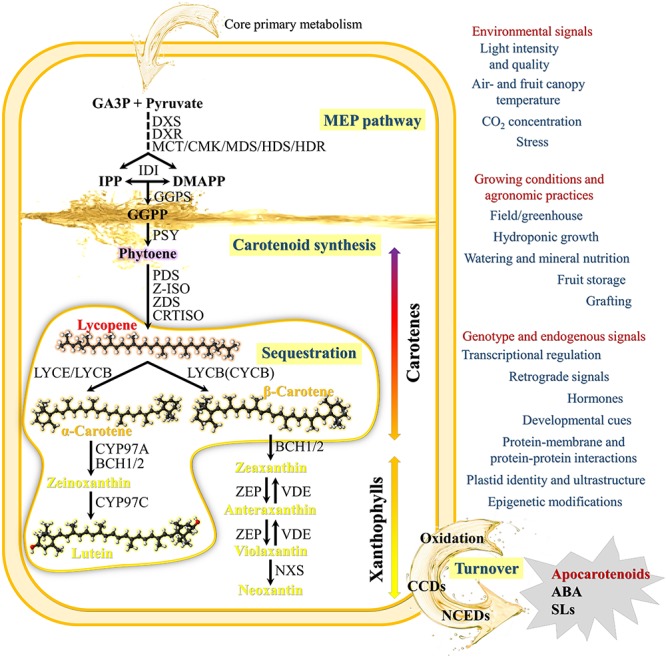
Schematic Carotenoid Metabolic Pathway and main factors affecting carotenoid synthesis and accumulation. The pathway shows the primary steps found in nearly all plant species. The synthesis of carotenoids from core primary metabolism initiates via the plastid-localized 2-*C*-methyl-D-erythritol 4-phosphate (MEP) pathway, leading to the production of isopentenyl diphosphate (IPP) and its isomer dimethylallyl diphosphate (DMAPP), central precursors of other isoprenoid metabolites including tocopherols, chlorophylls, quinones, gibberellins (GA), and monoterpenes. The first step of the MEP pathway involves the transketolase-type condensation of pyruvate and D-glyceraldehyde 3-phosphate (GA3P) to 1-deoxy-D-xylulose 5-phosphate (DXP) and is catalyzed by the enzyme DXP synthase (DXS). MEP is subsequently formed via an intramolecular rearrangement and reduction of DXP by the enzyme DXP reductoisomerase (DXR). MEP is then converted in IPP and DMAPP by a number of consecutive steps catalyzed by five independent enzymes: 2-*C*-methyl-D-erythritol 4-phosphate cytidyltransferase (MCT), 4-diphosphocytidyl-2-*C*-methyl-D-erythritol kinase (CMK), 2-*C*-methyl-D-erythritol 2,4-cyclodiphosphate synthase (MDS), 4-hydroxy-3-methylbut-2-en-1-yl diphosphate synthase (HDS), and 4-hydroxy-3-methylbut-2-enyl diphosphate reductase (HDR). IPP isomerase (IDI) catalyzes the isomerization of the relatively un-reactive (IPP) to the more-reactive DMAPP. Geranylgeranyl diphosphate (GGPP), the immediate C_20_ ubiquitous isoprenoid precursor in the synthesis of all plastid carotenoids, is generated by geranylgeranyl diphosphate synthase (GGPS) that catalyzes the condensation of three IPP and one DMAPP units. The first committed step in carotenoid biosynthesis is the condensation of two molecules of GGPP by phytoene synthase (PSY) to form the C_40_ carotenoid phytoene as a 15-*(Z)* isomer. Phytoene is converted to all-*trans*-lycopene by sequential desaturation and *Z-E* isomerization reactions. In plants, at least four enzymes are required: phytoene desaturase (PDS), ζ-carotene desaturase (ZDS), ζ-carotene isomerase (Z-ISO) and carotenoid isomerase (CRTISO). PDS/ZISO and ZDS/CRTISO constitute metabolic units involved in the steps catalyzing the synthesis of 9,9′-di-*(Z)*-ζ-carotene, and *all(E)*-lycopene, respectively (Fantini et al., 2013). Cyclization of lycopene with lycopene 𝜀- (LCYE) and β-cyclases (LCYB) is a crucial branch-point in carotenoid metabolism and generates carotenoid diversity distinguished by different cyclic end groups. In one branch, a single enzyme, lycopene β-cyclase (LCYB or CYCB in tomato fruits), introduces a β-ring at both ends of lycopene to form β-carotene in da sequential two-step reaction. The first dedicated reaction in the other branch, leading to lutein, requires both LCYE and LCYB to introduce one β- and one 𝜀-ring into lycopene to form α-carotene. α-carotene and β-carotene are further hydroxylated to produce xanthophylls (e.g., lutein and zeaxanthin). α-carotene is acted upon by β-ring hydroxylases (CYP97A and BCH1/2) to form zeinoxanthin, which is then hydroxylated by an 𝜀-ring hydroxylase (CYP97C) to lutein. β-carotene can be hydroxylated in a two-step reaction to zeaxanthin, with β-cryptoxanthin as an intermediate product by BCH1/2. Zeaxanthin epoxidase (ZEP) hydroxylates β-rings of zeaxanthin in two consecutive steps to yield antheraxanthin and then violaxanthin. Zeaxanthin can be epoxidized to violaxanthin, and a set of light- and dark-controlled reactions known as the xanthophyll cycle rapidly optimize the concentration of violaxanthin and zeaxanthin in the cell through the action of zeaxanthin epoxidase (ZEP) and violaxanthin de-epoxidase (VDE), respectively, via antheraxanthin. Violaxanthin is converted to neoxanthin by neoxanthin synthase (NXS), which represents the final step in the core carotenoid biosynthetic pathway. Catabolism of carotenoids proceeds by their cleavage by enzymes of the carotenoid cleavage dioxygenase family (CCDs) and 9-*(Z)*-epoxy-carotenoid dioxygenase family (NCEDs) to produce various apocarotenoids, phytohormones (ABA and SLs) and isoprenoid volatiles. In addition to enzymatic cleavage, oxidation via peroxidases/lipo-oxygenases or non-enzymatic photochemical oxidation of carotenoids have also evidenced especially in photosynthetic tissues under high light stress and suggested to mediate carotenoid homeostasis.

**FIGURE 2:**
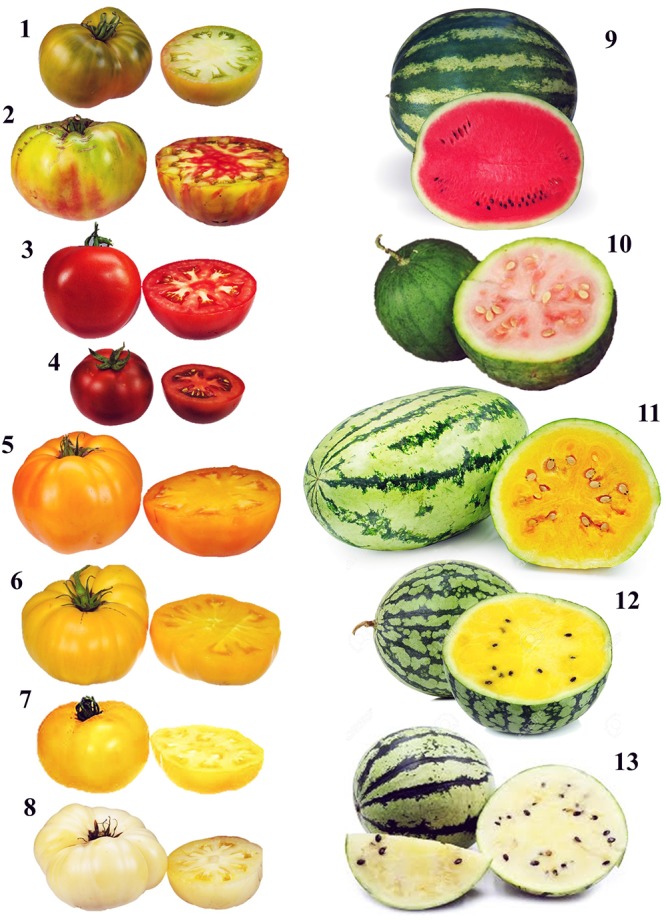
Example of color variability in tomato and watermelon. 1, Cherokee Green (green); 2, Ananas Noir (multicolor); 3, Primo Red (red); 4, Geronimo (dark red); 5, Black Velvet (orange); 6, Orange Slice (yellow); 7, Lemon Boy (bright yellow); 8, White Wonder (white); 9, Crimson Sweet (red); 10, Malali (salmon), 11, Tendersweet (orange); 12, Yellow Doll (yellow); 13, White Wonder (white).

Although in higher plants the biosynthesis of carotenoids occurs via the general isoprenoid pathway (Figure 1), their amount, composition and ratio are amazingly reliable in the photosynthetic tissues of different species, while large variation exists in non-green tissues even within the same crop (Yuan et al., 2015). In some fruits, the rate of carotenoid synthesis increases substantially during ripening alongside their hyper-accumulation into pigment-bearing sequestration sub-compartments, and results in dramatic changes in tissue colouration according to specific genetic programs, as well as in response to molecular, developmental and environmental stimuli. The ripe berries of tomato and the fleshy endocarp of watermelon peponides are clear examples of this variability, as a wide range of fruit colors and shades characterize both species (Figure 2).

Ordinary red tomato and watermelon cultivars appear to have a similar carotenoid biosynthetic pathway leading to the synthesis and accumulation of large amounts of lycopene as major fruit pigment (Tadmor et al., 2005). Lycopene is the linear carotene responsible of the distinctive color of both fruits at full ripeness and has aroused considerable interest as health-promoting phytochemical, because its dietary intake positively correlates with lowered risk of coronary heart disease, prostate, and lung cancers (Carluccio et al., 2016; Bruno et al., 2018; Rao et al., 2018).

In most plants, lycopene is an intermediate in the biosynthesis of other carotenoids and is cyclized at both ends by specific lycopene cyclase enzymes (LYCB/CYCB and LYCE) to α- and/or β-carotenes. However, in tomato and watermelon fruits, the downstream gene expression of *LYCB*/CYCB and *LYCE* is downregulated resulting in the break of the metabolic flux with the consequent accumulation of the upstream product (Lv et al., 2015; Enfissi et al., 2017). Lycopene accounts, in fact, for more than 85% of total carotenoids in many red-ripe tomato cultivars and for even higher percentages (>90%) in red-fleshed watermelons. In both fruits much lower concentrations of β-carotene (<10% and <5%, respectively) are typically found, while the content of other carotenes and xanthophylls is almost negligible (Tadmor et al., 2005; Perkins-Veazie et al., 2006; Tlili et al., 2011a; Liu et al., 2015).

### Fruit Concentration and Distribution

Genotype is a major determinant of the extent of variability in the content of carotenoids of ripe fruits in both tomato and watermelon. Lycopene and β-carotene levels in the range of 10–150 and 3.0–12.5 mg/kg fresh weight (fw), respectively, are common in ordinary red tomato cultivars; similarly, in most red-fleshed watermelon lines of commercial importance, lycopene varies between 30 and 70 mg/kg fw and β-carotene between 1.2 and 10.2 mg/kg fw (Perkins-Veazie et al., 2001, 2006; Ilahy et al., 2018). It is worthwhile mentioning that the mean lycopene concentration of watermelon (48.7 mg/kg fw) is about 40% higher than the mean for conventional red-ripe raw tomato (30.3 mg/kg fw). This indicates both species as comparable dietary sources of this powerful functional compound, although tomato and/or its numerous industrial products enter almost daily in the diet of most populations all around the world, while watermelon consumption is generally restricted to the summer season. Furthermore, a study on healthy subjects revealed that lycopene from untreated watermelon juice is just as bio-available as that from tomato juice subjected to heat, a treatment assumed to improve lycopene bioavailability (Edwards et al., 2003).

The introgression of spontaneous or induced color mutations is widely used to increase the levels and diversify the profile of carotenoids of tomato and watermelon and contributes to broaden the high variability characterizing these fruits. Many monogenic mutations affecting fruit carotenoid pigmentation have been isolated in tomato since the late 1940s and their molecular basis have been elucidated. Some of them trigger a deeper red colouration of the ripe fruits by increasing lycopene content. Several high-lycopene (HLY) tomato lines have been developed so far by introgression of *high-pigment* (*hp*) and *old-gold* (*og*) mutations (Ilahy et al., 2018). *Hp* tomato mutants all share a common phenotype characterized by enhanced plastid number and size, and consequential increased pigmentation of both unripe and ripe fruits, but they result from mutations of different genes. *Hp-1* and *hp-2* mutants carry single nucleotide alterations in the tomato *UV-damaged DNA-binding protein 1* (*DDB1*) and *DE-ETIOLATED1* (*DET1*) loci, respectively (Mustilli et al., 1999; Lieberman et al., 2004). Both mutations determine an exaggerated phytochrome-mediated response to light resulting in increased levels of carotenoids, mainly lycopene, and other antioxidant molecules (phenols, flavonoids, and vitamin C) in the ripe fruits, compared to their near isogenic wild-type counterparts (Liu et al., 2004; Levin et al., 2006). *Hp-3* mutants, instead, are not photomorphogenic, but harbor allelic mutations in the zeaxanthin epoxidase (*ZEP*) gene leading to a drastic reduction in the conversion of zeaxanthin to violaxanthin, and ultimately synthesis of ABA, whose deficiency is proposed to cause the enlargement of plastid compartment size by increasing their division rate (Galpaz et al., 2008). *Og* and *old-gold crimson* (*og^c^*), are both recessive null alleles of the *Beta* (*B*) locus coding for a tomato chromoplast-specific form of β-cyclase (CYCB) that increase ripe fruit lycopene concentration at expense of β-carotene. In *og* tomatoes, the lack of feedback inhibition by β-carotene (or one of its metabolites) might further increase the activity of the upstream enzymes during ripening resulting in lycopene hyper-accumulation, as suggested by Bramley (2002). Depending upon mutation, HLY red-ripe tomato lines contain variable amounts of lycopene and β-carotene, but usually higher (up to twofold) than the respective ordinary control cultivars (Ilahy et al., 2018).

Along with red cultivars, many white, yellow, and orange tomato heirloom or hybrid genotypes have been described and characterized for their diverse carotenoid profiles and contents. Most of these color variants are the results of mutations in single genes coding for enzymes involved in different steps of the carotenoid biosynthetic pathway. Some white and yellow tomato genotypes show a strong reduction (or even a complete lack) of carotenoids, due to a loss-of-function mutation of the *PSY-1* (known as *yellow-flesh*, locus *r*) gene. This determines a block in the first committed step of the biosynthetic pathway and results in the pale-yellow flesh, and more intensely yellow-colored skin phenotype typical of the ripe fruits (Lewinsohn et al., 2005b). Different orange cultivars were found to accumulate poly*(Z)*-lycopene (pro-lycopene), β-carotene or δ-carotene instead of *all(E)*-lycopene. Pro-lycopene accumulation in *tangerine* (*t*) tomatoes is due to a mutation in the *CRTISO* gene coding for a carotenoid isomerase catalyzing the desaturation of carotenoids from the poly*(Z)-* to the *all(E)*-configuration (Isaacson et al., 2002). The accumulation of β- or δ-carotene is, instead, the result of the upregulation of one of the two lycopene cyclase genes. In particular, the dominant *Beta* (*B*) mutation is responsible of the upregulation *CYCB* leading to the cyclization of lycopene to β-carotene, while *Delta* (*Del*) mutants result from an upregulation of *LCYE*, involved in the synthesis of δ-carotene (Ronen et al., 2000).

Like tomato, several deep red-fleshed watermelon cultivars have been bred. These lines show high lycopene contents ranging from 80 to over 120 mg/kg fw. Further, watermelon also exhibits a wide range of fruit color phenotypes [canary yellow, salmon yellow, orange, deep-red, and white (Figure 2)] supposed to result by the expression of orthologous of the *og*, *r*, *t*, *Del* and *B* tomato mutations (Tadmor et al., 2005; Zhao et al., 2013). However, this categorization, based exclusively on the HPLC carotenoid profiles, was shown to be often inconsistent. In the orange-fleshed watermelon cultivar NY162003, for example, β-carotene accounts for more than 99%, resembling an extreme case of tomato *B* mutation. Similarly, the deep-red HLY “Moon and Stars” cultivar has a phenotype corresponding to the *og* tomato mutant. However, the *CYCB* gene known to control colouration in tomato *B* and *og* mutants has not been detected in watermelon, thus the above-mentioned phenotypes are likely based on different molecular mechanisms (Bang et al., 2010; Lv et al., 2015). At this regard, Bang et al. (2007) proposed *LCYB* as the color-determining gene between canary yellow and red flesh varieties. The authors suggested that a single-nucleotide mutation in the *LCYB* gene introduced an amino acid replacement in the catalytic site, significantly impairing the activity of the enzyme with a consequent lycopene accumulation and appearance of the red phenotype. The active (wild) form of the enzyme is, instead, responsible for the canary-yellow phenotype. In yellow-fleshed watermelons, neoxanthin, violaxanthin, luteoxanthin and neochrome were found to be the predominant carotenoids indicating that, in the absence of an upstream blockage of lycopene cyclization, carotenoid metabolism proceeds rapidly toward the synthesis of xanthophylls (Bang et al., 2010; Lv et al., 2015). In watermelon, the enzymes carotenoid cleavage dioxygenases (CCDs) and 9-*(Z)*-epoxy-carotenoid dioxygenases (NCEDs), involved in the catabolic pathway, are candidate to play a fundamental role in fruit color determination. In fact, although, the up-/down-regulation of the carotenogenic genes direct the biosynthetic flow to specific carotenoids, the regulation of the catabolic genes affects the degradation/storage of the end-products. Accordingly, a high expression of *NCED* genes, particularly in the advanced phases of fruit development and ripening was demonstrated in pink, yellow, and white watermelon lines, leading to the cleavage of at least part of the synthesized xanthophylls and contributing to the observed reduced carotenoid content of these genotypes (Kang et al., 2010). Similar results were described in tomato, where fruit-specific RNAi-mediated suppression of *SINCED1* increased the accumulation of upstream compounds in the carotenoid pathway (Sun et al., 2012).

In non-red watermelon lines, the reduced carotenoid content has been, also, attributed to a generalized reduced transcription of most genes involved the biosynthetic pathway. For example, the transcript levels of almost all carotenogenic genes (except *LCYB* and *ZEP*) of the white genotype “ZXG507” were found lower than those of all the assayed colored cultivars at the same developmental stage, while the *NCED1* transcripts increased dramatically in the late ripening stages (Lv et al., 2015).

Fruit dimensions also seem to affect carotenoid content in tomato, with small fruited red-ripe cherry tomatoes generally showing higher lycopene and β-carotene contents than cluster, elongate and salad types (Leonardi et al., 2000; Lenucci et al., 2006). Instead, no systematic reports on different sized red watermelon genotypes are currently available. Nevertheless, it has been generally reported that seedless mini watermelon cultivars have higher amounts of lycopene (>50.0 mg/kg fw) than seeded open pollinated large sized ones (Perkins-Veazie et al., 2001). Furthermore, ploidy, often associated to changes in fruit dimensions, was found to affect lycopene content. Polyploids, especially triploids, had higher levels of lycopene than their diploid progenitors, and triploids tended to contain more lycopene than tetraploids (Liu et al., 2010).

Fractionate analyses of the peels, pulp and seeds isolated from red-ripe berries of different tomato cultivars revealed substantial differences among fractions. The peels showed up to fivefold more lycopene and β-carotene than pulp, while seed fractions were almost devoid of both carotenoids (Ilahy and Hdider, 2007; Chandra and Ramalingam, 2011; Chandra et al., 2012; Ilahy et al., 2016a). Similarly, topological differences on lycopene concentration were reported by Tlili et al. (2011b) in the full-ripe endocarp of different red watermelon cultivars, with the stem-end and the heart areas showing significant higher values than the blossom-end and the peripheral areas, and rind being completely devoid of the linear carotene, suggesting a spatial regulation of carotenoid metabolism.

Besides the genetic potential, many authors highlighted a large variability in lycopene and β-carotene concentrations and ratios in dependence of environmental factors, agro-technical processes, ripening stage, harvest and postharvest manipulations both in tomato and watermelon fruits (Abushita et al., 2000; Dumas et al., 2003; Perkins-Veazie and Collins, 2004; Lenucci et al., 2009; Siddiqui et al., 2018).

### Accumulation Factors and Regulation

In tomato, light radiation intensity and quality, air- and fruit canopy temperature, CO_2_ concentration and growing system (irrigation, fertilization, grafting, etc.) have been demonstrated to significantly affect fruit carotenoid concentration and confer prospects for enhancing their accumulation without resorting to metabolic engineering (Pogonyi et al., 2005; Brandt et al., 2006; Dorais, 2007). Light and circadian rhythm are known to alter the expression of nearly all MEP genes and several carotenoid synthesis and catabolism genes (Liu et al., 2004; Cordoba et al., 2009). Tomato fruits subjected to high irradiance and high temperature have demonstrated an increased metabolism of carotenoids, probably in relation to the protective role of carotenoids against the resulting oxidative stress, and/or the capacity of dissipating excess absorbed energy in the xanthophyll cycle (Cocaliadis et al., 2014). Oxidative stress in tomato fruit increases co-ordinately with fruit ripening and reaches a peak at the final stages thus triggering metabolic changes and fruit softening (Jimenez et al., 2002). It is also known that red-light and far-red light, respectively, stimulate and inhibit carotenoid accumulation in tomato by a mechanism likely involving phytochrome receptors (Alba et al., 2000; Giovannoni, 2001; Schofield and Paliyath, 2005). In ripening tomato, the content of lycopene and β-carotene positively correlated with an increase of photosynthetically active radiation (PAR) and more precisely with exposure to blue-light (Gautier et al., 2004).

Light also has a major role in modulating the developmental programs determining plastid identity and ultrastructure. Plastid type and sub-organelle compartmentalization not only influence carotenoid profile and storage capacity but also play a central role in controlling the activity of PSY and other enzymes of the metabolic pathway. During tomato fruit ripening, light stimulates the differentiation of chromoplasts and induces carotenoid biosynthesis, while in non-photosynthetic tissues of carrot roots, it promotes chloroplasts differentiation instead of chromoplasts. Furthermore, tomato fruit carotenoid biosynthesis seems adjusted to actual ripening progression by a light-dependent mechanism involving the endogenous shade signaling components of chloroplasts. During chloroplast-to-chromoplast transition the controlled chlorophyll degradation reduces the self-shading effect and promotes the turnover of a transcription factor (*Pif1a*) that directly repress PSY1 expression, boosting carotenoid biosynthesis as ripening progress (Llorente et al., 2017). As far as we know, there are no specific reports on the effects of light on the biosynthesis of carotenoids in watermelon fruit tissues. Nevertheless, light intensity, temperature, and irrigation were reported to alter lycopene content by 10–20% in ripe watermelon fruits (Perkins-Veazie et al., 2001; Leskovar et al., 2004).

In tomato fruits, lycopene biosynthesis is strongly inhibited at temperatures below 12°C and completely blocked above 32°C (Dumas et al., 2003). An increase of lycopene cyclization to β-carotene, resulting in a decrease of lycopene content, was observed in fruits exposed to temperatures above 35°C (Hamauzu et al., 1998). Elevated CO_2_ levels also decrease significantly the lycopene content in greenhouse-grown tomatoes (Helyes et al., 2011). The authors proposed that the elevated air CO_2_ concentration might support the plants to cope better with environmental stresses, reducing the need of activating the stress response systems, including the synthesis of carotenoids. Postharvest ripening of tomato fruits at high CO_2_ atmospheres significantly prevented the rise in ethylene (ET) production, and slowed down lycopene biosynthesis and chlorophyll degradation, suggesting an alternative mechanism by which CO_2_ can indirectly affect carotenoid accumulation (Sozzi et al., 1999). Field experiments showed that a shift from 20 to 37°C of on-vine watermelon fruits did not result in color reduction as in tomato, indicating that, in this thermic range, lycopene synthesis is not affected by temperature (Vogele, 1937). Additionally, watermelon stored at 21°C gained 11–40% in lycopene and 50–139% in β-carotene, indicating active carotenoid biosynthesis as observed in tomato during off-vine ripening, whereas, little change in carotenoid content was reported at temperatures below 13°C (Perkins-Veazie et al., 2006).

Growing systems also affect the carotenoid content of tomato and watermelon. Field-grown tomatoes generally have higher levels of lycopene than greenhouse-grown tomatoes, in which it ranges between 1 and 108 mg/kg fw (Sahlin et al., 2004). As far as we know, no literature is available at this regard for watermelon. The effects of mineral nutrition on lycopene levels in tomato and watermelon are yet no conclusive, as the response is strongly dependent on rate, genotype, growing conditions and growth stage. In tomato, potassium (K) and/or phosphorous (P) supplementation in either soil or soilless (hydroponic) cultivation significantly increased, up to 30%, lycopene and total carotenoid content of ripe fruits (Dumas et al., 2003; Serio et al., 2007; Almeselmani et al., 2009; Bidari and Hebsur, 2011). Conversely, little or no increase in lycopene with increased soil K or P rates was reported in tomato and watermelon fruits by Fontes et al. (2000) and Perkins-Veazie et al. (2003). The ratio of K and nitrogen (N) has been also shown to affect the content of total carotenoids and lycopene of hydroponically grown tomatoes (Kaur et al., 2018). K concentration has been proposed to play an indirect role in the process of carotenoid biosynthesis in tomato by activating several of the enzymes regulating the metabolism of carbohydrates and 2-*C*-methyl-D-erythritol 4-phosphate (MEP) pathway, leading to an increase of the precursors of isopentenyl diphosphate (IPP) (Fanasca et al., 2006).

The response to water amount and quality on lycopene content depends on crop and germplasm. Some tomato cultivars showed a decrease of lycopene with decreased soil water, while in other it was increased. Salinity (0.25% NaCl w/v) significantly improved lycopene content of tomatoes, but the response was genotype dependent (De Pascale et al., 2001; Krauss et al., 2006). Evidence suggests that osmotic and/or salt stress cause ET synthesis, which is a main positive regulator of lycopene synthesis in tomato (Wu and Kubota, 2008).

No significant differences in lycopene content were reported in watermelon irrigated at different regimes, while it positively correlated with salinity increase (Ban et al., 2014). Conversely, a decrease in the expression of *PSY*, *PDS*, *ZDS*, *LCY-β* genes was reported at increasing salt concentrations (between 0 and 200 mM NaCl) by Cheng et al. (2015).

Grafting is used widely with tomato and watermelon to improve water and mineral nutrition of plants, enhance the tolerance to soil-borne diseases (e.g., *fusarium* wilt) and abiotic stress, as well as to increase yield and fruit quality (Kyriacou et al., 2017). Complex interactions between rootstock type, growing season, salinity, and genotype on carotenoid concentration, particularly lycopene, have been reported in both vegetable crops, leading to conflicting results (Fekete et al., 2015; Marsic et al., 2018).

Despite the common trait of synthesizing and accumulating lycopene, red tomato and watermelon show similarities and differences in the mechanisms that implement and regulate the process. First, a polyphyletic origin of the trait has been proposed based on the different fruit color of the putative wild ancestors of the domesticated tomato and watermelon. *Solanum pimpinellifolium* L., the progenitor of modern tomato, has intensely colored red berries (Rick, 1995), *C. lanatus* var. *citroides* (L.H. Bailey) Mansf., the supposed ancestor of watermelon, has, instead, white fleshed fruits (Navot and Zamir, 1987), suggesting that the genetic changes resulting in the red-flesh trait occurred after watermelon had been domesticated (Tadmor et al., 2005).

A further manifest difference between tomato and watermelon lies in the color transition during fruit ripening, which is the result of different developmental programs of chromoplasts differentiation. Ripening tomatoes undergo a marked green-to-red color change, due to the interconversion of chromoplasts from pre-existing photosynthetic chloroplasts (Suzuki et al., 2015). Green-unripe tomato fruits show, in fact, the typical carotenoid profile of photosynthetically active tissues (Bramley, 2002). In ripening tomato fruits, chloroplast-to-chromoplast interconversion is marked by a burst of lycopene synthesis and its massive accumulation as large crystals within membrane-shaped structures, accompanied by the simultaneous breakdown of thylakoid membrane, the *de novo* formation of membranous carotenoid-sequestrating substructures within chromoplasts, and the increase in the number and size of plastoglobules (Simkin et al., 2007; Bangalore et al., 2008). The white flesh of young watermelon fruits contains, instead, only trace amounts of carotenoids because chromoplasts differentiate from non-photosynthetic plastids, possibly directly from undifferentiated proplastids (Wang et al., 2013). Ordinary and HLY tomatoes showed a similar pattern of change in lycopene and β-carotene during ripening, with a sharp increase in the synthesis of these carotenoids during the transition from the green to the breaker stage of ripening, suggesting that the mechanisms regulating the process are conserved among genotypes (Hdider et al., 2013). A similar trend was reported in different commercial and newly HLY developed watermelon lines by Tlili et al. (2011a), with the burst in carotenoid biosynthesis occurring between the white-pink and the pink stages of ripening. As previously told, the regulation of chromoplast biogenesis plays a crucial role in providing a site not only for active carotenoid biosynthesis but also for carotenoid storage. In chromoplasts, various lipoprotein substructures (e.g., globules, crystals, membranes, fibrils, and tubules) sequester carotenoids. Based on the pigment-bearing sequestration substructures, chromoplasts are classified into five major types as globular, crystalline, membranous, fibrillar, and tubular (Egea et al., 2010; Li and Yuan, 2013; Schweiggert and Carle, 2017). Usually more than one kind of chromoplasts co-exists in a species. Tomato has globular chromoplasts characterized by abundant plastoglobules. Crystalline chromoplasts typically over-accumulate lycopene and β-carotene as red/orange crystals, and are abundant in tomato, and watermelon (Jeffery et al., 2012; Zhang et al., 2017).

Moreover, tomato fruits are climacteric, thus ripening processes, including lycopene synthesis and accumulation, are dependent on ET; watermelon, instead, is non-climacteric. These differences are reflected by the mechanisms controlling the biosynthesis and accumulation of carotenoids in the two crops.

The carotenoid metabolism and accumulation in tomato and watermelon fruits is temporally and spatially regulated by systematic and sophisticated mechanisms and is dependent upon several factors besides the modulation of the pathway gene expression (Nisar et al., 2015; Yuan et al., 2015). Plants have evolved, in fact, diverse strategies to regulate, at multiple levels, carotenoid metabolism in response to developmental programs, environmental factors, and metabolic signals. Transcriptional regulation certainly provides the first layer of control in the biosynthesis and accumulation of specific carotenoids during fruit ripening of both species. The transcription of many genes of the MEP, carotenoid biosynthetic and catabolic pathways have been found to be up- or down-regulated during tomato and watermelon fruit ripening (Apel and Bock, 2009). In red conventional cultivars of tomato and watermelon the transcription of precursor (upstream) carotenogenic genes encoding for specific fruit isoforms of DXS, GGPS, PSY, PDS, ZDS, CRTISO are up-regulated during ripening and parallel the synthesis and accumulation of lycopene (Fraser et al., 2002; Grassi et al., 2013). In tomato, plastid isopentenyl diphosphate isomerase (IDI) was shown to play an important function in carotenoid biosynthesis, thus highlighting its role in optimizing the ratio between IPP and dimethylallyl diphosphate (DMAPP) as precursors for different downstream isoprenoid pathways (Pankratov et al., 2016). The expression of downstream *LCY-E* and *LCY-B* genes is, instead, dramatically decreased, or maintained at low levels, during ripening (Ronen et al., 1999; Tadmor et al., 2005; Kang et al., 2010; Zhang et al., 2013). Cyclization of lycopene is a key regulatory branching point of carotenogenesis in both crops, since *LCYB/CYCB* and *LCYE* are involved in channeling lycopene into the downstream pathway. In the red-fleshed watermelon cultivar Dumara, for example, a constant low expression level of both *LCYB* and *LCYE* was reported and related to the biogenesis of chromoplasts from non-photosynthetic plastids. In fact, in contrast to tomato, no chloroplast-to-chromoplast transition occurs in watermelon flesh, thus the metabolic flux toward cyclic carotenes and xanthophylls, which are present in significant quantities in the purified chloroplasts of unripe tomatoes (Lenucci et al., 2012; Ilahy et al., 2014), can be permanently maintained at low levels during the entire process of fruit development and ripening. Nevertheless, significant differences in *LCYB* expression patterns were reported among red watermelon genotypes and between these and non-red watermelons (Guo et al., 2015). Besides, in the cultivar Dumara, the high expression levels of downstream *BCH* and *ZEP*, whose expression increased early during fruit ripening and remained stable over time, may help maintain the amounts of γ- and β-carotene at low levels as intermediate metabolites (Grassi et al., 2013). Accordingly, the lack of both zeaxanthin and violaxanthin, products of *CHYB* and *ZEP* activities, in the watermelon carotenoid profiles at any stage of ripening supports the hypothesis of their rapid turnover toward the synthesis of phytohormones and/or other carotenoid derived signaling molecules (Kang et al., 2010). The steady-state level of carotenoids is, in fact, the result of the equilibrium between synthesis and degradation (Li and Yuan, 2013). Thus, the catalytic activity of CCDs is critical in regulating carotenoid accumulation. In tomato, *CCD1* expression has been associated with the emission of isoprenoid volatiles, including neral [*(Z)*-citral], geranial [*(E)*-citral], and farnesyl acetone, whose production is correlated to carotenoid levels (Klee and Giovannoni, 2011; Ilg et al., 2014). Similarly, different members of the NCED family have been shown to increase during tomato and watermelon ripening.

In most plants, phytohormones and transcription factors operate in concert to finely tune the expression of carotenogenic genes (Zhong et al., 2013). Phytohormones, including ET, auxin, ABA, gibberellic acid (GA), jasmonic acid (JA) and brassinosteroids (BR), as well as non-enzymatic oxidation carotenoid-derived compounds have been proposed to directly or indirectly regulate ripening and carotenoid accumulation in tomato fruits (Ma et al., 2009; Havaux, 2014; Liu et al., 2015; Su et al., 2015; Cruz et al., 2018). Among them, ET plays a central role. In tomato fruits, the onset of ripening is triggered by a dramatic increase in ET production, correlating with the rapid accumulation of β-carotene and lycopene, and the expression of *SlPSY1* and *SlPDS* is dependent on ET (Marty et al., 2005). Following the application of various hormone-like substance to tomato fruits at the mature-green ripening stage, Su et al. (2015) demonstrated that auxin application, as indole-3-acetic acid (IAA), retards tomato ripening by affecting a set of key regulators, such as *Rin*, ET and ABA, and key effectors, such as genes for lycopene and β-xanthophyll biosynthesis and for chlorophyll degradation, thus suggesting that carotenoid accumulation during tomato fruit ripening is modulated by the IAA-ET balance. Recently, ET and auxin have been proposed to be involved as part of the light signaling cascades controlling tomato fruit metabolism, providing a new crosstalk between light signaling, plant hormone sensitivity and carotenoid metabolism in ripening tomato fruits (Cruz et al., 2018). At the best of our knowledge, the role of phytohormones in carotenoid biosynthesis has not been thoroughly investigated in watermelon fruits. However, although ET biosynthesis is not essential for watermelon fruit ripening, varying patterns of production have been reported, indicating that ET and/or a modulated sensitivity to the hormone might participate in physiological changes during watermelon fruit development (Perkins-Veazie et al., 1995). Indeed, many non-climacteric fruits, including watermelon, are highly sensitive to exogenous ET (Chen et al., 2018).

Recently, ABA has been shown to be involved in the regulation of watermelon fruit ripening, but if it is directly involved in carotenoid synthesis and accumulation is still not known (Wang et al., 2017).

Several ripening-related regulatory factors, particularly those involved in ET production and sensing, were found to finely control the carotenoid levels in tomato fruits (Cazzonelli and Pogson, 2010; Enfissi et al., 2010; Liu et al., 2015). These include the *RIPENING INHIBITOR (RIN-MADS)*, *TOMATO AGAMOUS-LIKE 1 (TAGL1)*, *ETHYLENE RESPONSE FACTOR 6* (*ERF6)*, *APETALA2a (AP2a)*, *MC*, *NON-RIPENING* (*NAC-NOR*), *DE-ETIOLATED1* (*DET1)*, *UV-DAMAGED DNA BINDING PROTEIN1* (*DDB1)*, *CULLIN-4 (CUL4)*, *COLORLESS NON RIPENING* (*CNR*), *GOLDEN 2-LIKE (GLK2)*, *HB-1*, *UNIFORM RIPENING (U)/GLK2*, *FRUITFULL 1*/*2* (FUL1/TDR4; FUL2/MBP7), and *B-BOX20* (*BBX20*) transcriptional regulators (Vrebalov et al., 2002; Manning et al., 2006; Lin et al., 2008; Itkin et al., 2009; Chung et al., 2010; Powell et al., 2012; Pan et al., 2013). In watermelon although homologous genes of most tomato transcription factors have been identified their transcription often showed different levels than in tomato, suggesting that while a common set of metabolic and regulatory genes is conserved and influences carotenoid accumulation during development and ripening, specific regulatory systems may differ possibly related to the different ripening physiologies of climacteric and non-climacteric fruits (Grassi et al., 2013).

Transcriptional regulation is not the only regulatory system directing carotenoid biosynthesis and accumulation in plants; post-translational and epigenetic mechanisms (Zhong et al., 2013; Arango et al., 2016) provide other adaptive layers of control. However, there is a great lack of knowledge about the identity of these processes both in tomato and watermelon (Lu and Li, 2008). Protein-membrane and protein–protein interactions, as well as feedback and feedforward control mechanisms (Luo et al., 2013; Chayut et al., 2017; Enfissi et al., 2017) have been proposed to modulate the levels and activities of carotenogenic enzymes in different species, including tomato. A tomato STAY-GREEN protein, *SlSGR1*, was shown to regulate lycopene accumulation during fruit ripening by directly inhibiting PSY1 activity. The formation of membrane-bound multi-enzyme complexes acting in sequence (metabolons) also facilitates metabolite channeling to drive flux toward completion (Ruiz-Sola et al., 2016). In tomato, the levels of different carotenoid metabolites exert a feedback and feedforward regulation of carotenoid pathway enzymes, acting predominantly at the level of PSY1 and cyclases (Kachanovsky et al., 2012; Enfissi et al., 2017). Light signals mediated by fruit-localized phytochromes as well as the physical sequestration of carotenoids in plastid sub-compartments from downstream enzymes have been also reported to affect the activity of the enzymes involved in carotenoid metabolism (Nogueira et al., 2013; Gupta et al., 2014). The extent to which parallel mechanisms operate in watermelon fruits is still unknown, but highly probable.

## Phenolics in Tomato and Watermelon Fruits

### Chemical Features and Functions

**FIGURE 3:**
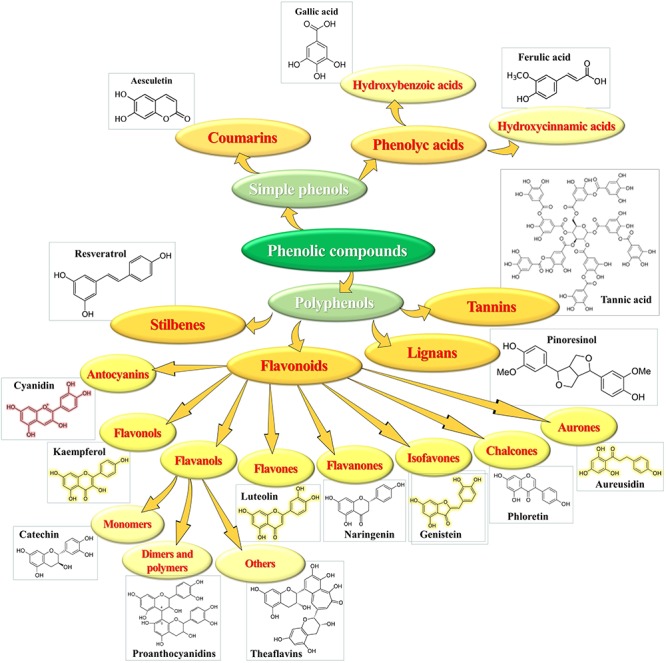
Chemical structure and classes of phenolic compounds.

Phenolics represent an important class of secondary metabolites broadly distributed in the plant kingdom, characterized by the presence of mono- or poly-hydroxylated aromatic rings (Dai and Mumper, 2010). Tens of thousands of diverse molecules, with the number incessantly increasing, ranging from hydrophilic, lipophilic to insoluble structures, have been identified in plants. Some occur as low molecular-weight monomers, other are dimerized, polymerized or even highly polymerized to large complex compounds. Based on the number of C-atoms and basic arrangement of carbon skeletons in their chemical structure, phenolics are classified in groups and sub-groups (Figure 3) with different characteristics and distribution (Alu’datt et al., 2017).

The complexity of functions of phenolics within plants is still poorly understood, however, fundamental roles as antioxidants, structural polymers, coloring pigments, chemo-attractants or repellents to pollinators or pests, UV-screens, signaling compounds in symbiosis initiation and plant–microbe interactions and defense weapons against aggressors have been suggested (Dai and Mumper, 2010). Besides, phenolics are responsible of various organoleptic traits in fruits and vegetables and have stimulated substantial attention as health-promoting compounds because of their antioxidant, anti-inflammatory, anti-allergic, anti-atherogenic, anti-thrombotic and anti-mutagenic effects (Cory et al., 2018; Shahidi and Yeo, 2018). The dietary intake of phenolics positively correlates to a reduced incidence of many chronic pathologies, including cardiovascular, neurodegenerative and neoplastic diseases. They seem also involved in the modulation of the human immune system by affecting the proliferation of white blood cells and the production of cytokines or other defense factors (Olivares-Vicente et al., 2018). Accordingly, breeders, growers, and processors are increasingly searching for tools to improve the profile and enhance the content of specific phenolics in newly released elite cultivars to respond for the growing interest of consumers for high quality fruits and vegetables.

### Biosynthesis

**FIGURE 4:**
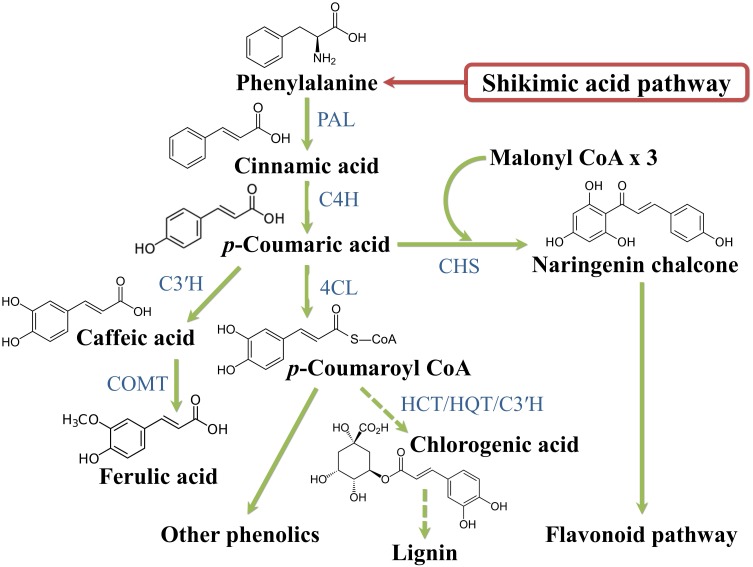
Phenylpropanoid biosynthetic pathway. 4CL, 4 coumarate CoA ligase; C3’H, *p-*coumaroyl ester 3 hydroxylase; C4H, cinnamate 4 hydroxylase; CHS, chalcone synthase; COMT I, caffeic/5-hydroxyferulic acid *O*-methyltransferase; HCT, hydroxycinnamoyl transferase; HQT, hydroxycinnamoyl CoA:quinate hydroxycinnamoyl transferase; PAL, phenylalanine ammonia lyase.

Plant phenolics arise biogenetically from a complex network of routes based principally on the shikimate/phenylpropanoid pathway or the “polyketide” acetate/malonate pathway (Cheynier et al., 2013). The shikimate pathway converts carbohydrates in the aromatic amino acids phenylalanine and tyrosine, the main substrates for the synthesis of hydroxycinnamic (*p*-coumaric, caffeic, ferulic and sinapic) acids (Figure 4). The limiting reaction that interlocks the primary and secondary metabolisms is catalyzed by phenylalanine ammonia lyase (PAL) that deaminates phenylalanine to cinnamic acid. Cinnamic acid is hydroxylated at the C4-position by the enzyme cinnamate 4-hydroxylase (C4H) to *p*-coumaric acid that, in turn, receives another hydroxyl at the C3-position by the activity of *p*-coumaroyl ester 3-hydroxylase (C3′H) to caffeic acid. Ferulic acid is biosynthesized from caffeic acid by the action of the enzyme caffeate *O*-methyltransferase (COMT). *p*-Coumaric acid can also be directly produced from tyrosine deamination, though this reaction, catalyzed by tyrosine ammonia lyase (TAL), is barely used by plants. *p*-Coumarate is converted to its CoA ester by the 4-coumarate:CoA ligase (4CL); this step is a central point of branching of the phenylpropanoid pathway (Cheynier et al., 2013).

**FIGURE 5:**
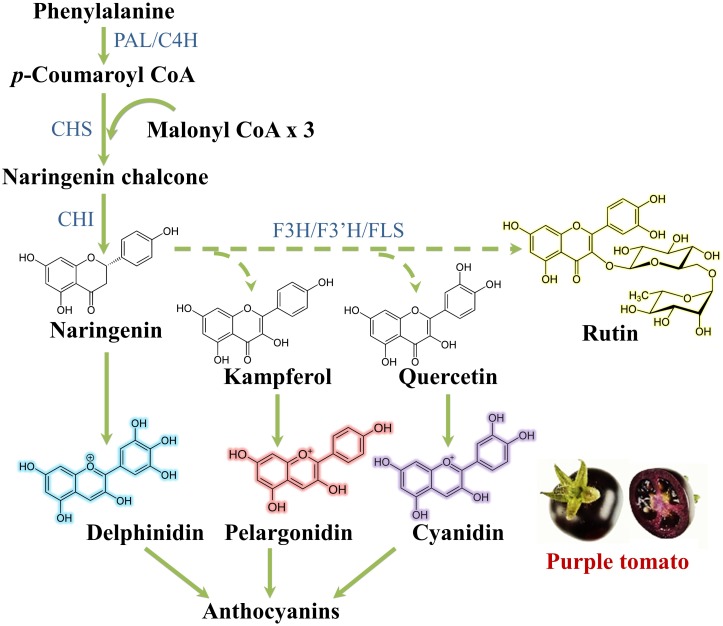
Flavonoid biosynthetic pathway. C4H, cinnamate 4 hydroxylase; CHI, chalcone isomerase; F3H, flavanone 3-hydroxylase; F3′H, flavanone 3′-hydroxylase; FLS, flavonol synthase; PAL, phenylalanine ammonia lyase.

Regarding flavonoid synthesis (Figure 5), one aromatic ring and its side chain arises from phenylalanine, while the other arises from acetyl-CoA via the acetate/malonate pathway: the *p*-coumaroyl-CoA reacts with three molecules of malonyl-CoA under the action of the key enzyme chalcone synthase (CHS) to produce naringenin chalcone. This is prone to isomerization, hydroxylation and reduction/oxidation reactions to give primary aglycone flavonoids. The action of glycosyltransferases, methyltransferases and acyltransferases further increases the heterogeneity of flavonoids (Saltveit, 2009).

### Fruit Concentration and Distribution

Ordinary red tomato and watermelon cultivars appear to have similar phenolic and flavonoid levels and, likely, analogous regulation mechanisms leading to the synthesis and accumulation of significant amounts of these compounds during ripening. The total phenolic content of different red tomato and watermelon cultivars is in the range 166–770 and 137–260 mg Gallic Acid Equivalent (GAE)/kg fw, respectively, with flavonoids [40–260 and 111–176 mg Rutin Equivalents (RE)/kg fw] substantially contributing to the total (Slimestad et al., 2008; Tlili et al., 2010, 2011a,b; Choudhary et al., 2015; Ilahy et al., 2018). Nevertheless, phenolic concentrations and profiles are strongly dependent on genotype, ripening stage, environmental factors, as well as pre- and post-harvest manipulations (Dumas et al., 2003; Ilahy et al., 2016b, 2018). This variability was confirmed also in HLY tomato cultivars, showing higher levels of phenolics and flavonoids than the ordinary tomato cultivars.

Besides, phenolic concentrations vary widely within the different fruit fractions (Ilahy et al., 2016a). Tomato peels showed 2–4 and 1.4–2.3 times higher total phenolic and flavonoid contents with respect to pulp. Tlili et al. (2011b) also evidenced a significant variation between the stem-end, blossom-end, heart and peripheral areas of ripe watermelon fruits in the concentrations of phenols and flavonoids.

Based on multiple analytical methods, Slimestad and Verheul (2009) reported the presence of 114 different phenolic molecules in ordinary red tomato fruits. These included mainly phenolic acids and flavonoids, indicated as the most represented groups in the ripe fruits. Phenolic acids contribute more than 70% to the total phenolics of red-ripe tomato fruits. They are distributed in the exocarp (peel), mesocarp and endocarp tissues primarily as chlorogenic acid (Moco et al., 2007), though the presence of *p*-coumaric, caffeic, ferulic, and sinapic acids has also been reported (Long et al., 2006). Flavonoids comprise mainly the flavanone naringenin and its isomer chalconaringenin (46%), followed by the flavonols quercetin (39%), myricetin (9%) and kaempferol (6%) (Haytowitz et al., 2018). Rutin (quercetin-3-*O*-rutinoside) is the major glycosylated flavonoid in ripe tomato fruits, although the flavonol quercetin 3-*O*-(2″-*O*-β-apiofuranosyl-6″-*O*-α-rhamnopyranosyl-β-glucopyranoside) and the dihydrochalcone phloretin 3′,5′-di-*C*-β-glucopyranoside often show equivalent levels. More than 95% of flavonols (rutin and kaempferol) is in the peels (Stewart et al., 2000; Ilahy et al., 2016a), where concentrations up to 1840 mg/100 g dry weight (dw) of the stilbenoid phytoalexin resveratrol and related glycosides have been detected, with little change during fruit ripening and a maximum at 4 weeks post breaker (Ragab et al., 2006). Besides, it has been reported that specific flavonoid molecules may be synthesized in the tomato peel under particular light conditions, suggesting an important role of irradiance quality and intensity in determining the level and the profile of phenolic compounds in fruits (Slimestad and Verheul, 2009).

While tomatoes have the genes for producing anthocyanins, they are typically not expressed in the fruit resulting in a negligible concentration of these health-promoting phenolic pigments in fruits. Recently, a “purple” tomato, highly enriched with anthocyanins, was produced by the ectopic expression of two selected transcription factors from snapdragon. The content of anthocyanins in the whole fruit was about 5.2 g/kg dw with little difference between pulp and peels. Petunidin-3-(*trans*-coumaroyl)-rutinoside-5-glucoside and delphinidin-3-(*trans*-coumaroyl)-rutinoside-5-glucoside were the most abundant anthocyanins, contributing up to 86% to the total. These fruits were found to extend the life of cancer-susceptible mice, suggesting that they have additional health-promoting effects (Su et al., 2016).

Luteolin and chlorogenic acid are the main phenolics detected in watermelon fruits (Fu et al., 2011). Interestingly, a combination of both molecules is regarded as prospective treatment against the inflammatory propagation in patients affected by rheumatoid arthritis (Lou et al., 2015). Abu-Hiamed (2017) evaluated the levels of flavonoids in watermelon external and internal pulp, as well as in the rind fraction. Rutin (1.66 mg/100 g fw) was detected exclusively in the internal pulp, while any of the tested fractions revealed the presence of quercetin and kaempferol, even though USDA flavonoid database reports the presence of kaempferol in a concentration ranging between 0 and 1.81 mg/100 g fw (Haytowitz et al., 2018). Chlorogenic, vanillic and sinapic acids are, instead, the main phenolics found in the rind of watermelon fruits (Mushtaq et al., 2015).

Extensive variability in phenolics and flavonoids levels has been highlighted during tomato and watermelon ripening. Ilahy et al. (2011) evidenced a complex and genotype-dependent pattern of changes in the amount of total phenolics and flavonoids among different cultivars of tomatoes, including both ordinary and HLY *hp-2* lines. The total phenolic content in the tomato cultivar HLY18 peaked (310 mg GAE/kg fw) at the orange-red stage, whereas cultivar HLY13 exhibited two consecutive peaks at the green and orange–red stages (223 and 240 mg GAE/kg fw, respectively). However, at the same ripening stages, the traditional cultivar Rio Grande showed the lowest total phenolics content (113 and 138 mg GAE/kg fw, respectively). Besides, flavonoid concentration peaked at the green-orange ripening stage in both HLY cultivars, but at the green stage in cultivar Rio Grande. Although the content of flavonoids was unchanged at advanced ripening stages, their levels were higher in HLY cultivars than in the traditional tomato cultivar Rio Grande regardless the stage of ripening.

Tlili et al. (2011a) focused on the dynamic of total phenols and flavonoids of different field grown watermelon cultivars (Crimson sweet, Dumara Giza, P503 and P403) throughout the ripening stages. Total phenols and flavonoids changed significantly during ripening. Crimson Sweet, Dumara and P503, P403 and Giza exhibited a peak of phenols at the red-ripe stage. Regarding flavonoid, a linear increase was observed during ripening of cultivar Crimson Sweet with more complex dynamic of change for the other cultivars.

### Accumulation Factors and Regulation

Being massively involved in signaling and response processes, the metabolism of phenolics in plants is severely affected by exogen stimuli (Rivero et al., 2001). Environmental factors have a strong influence over genotype. Fully ripe watermelon fruits grown in Southern Italy had 77–121% and 33–74% higher levels of phenols and flavonoids than those, of the same cultivars, grown in the North of Tunisia following identical agronomic procedures (Tlili et al., 2011a). Watermelon fruit quality is also prone to the influence of pre-harvest procedures and agro-technical processes such as irrigation, fertilization, grafting, and growing method (conventional/organic farming). Rivero et al. (2001) found that thermal stress induces a massive phenolic accumulation in tomato and watermelon fruits by activating the biosynthetic and inhibiting the oxidation pathways. This mechanism operates below 15°C to overcome chilling stress in watermelon and above 35°C in tomato to counteract heat stress and can be regarded as an acclimation response to extreme temperatures.

Storage conditions significantly affect tomato phenolics determining an increase or decrease depending on genotype, pre- and post-harvest handling (Ilahy et al., 2018). Nevertheless, some procedures have been proposed to preserve or increase the levels of total phenols during tomato storage including brassinolide, high-voltage electrostatic field, direct-electric-current and delactosed whey permeate treatments (Dannehl et al., 2011; Aghdam et al., 2012; Ilahy et al., 2018). Studies on the effect of postharvest procedures on the phenolic and flavonoid content of watermelon are lacking. Nevertheless, Perkins-Veazie and Collins (2004) reported possible alteration of the general antioxidant component by temperature and storage.

At the gateway from primary metabolism, PAL is known to play a pivotal role in the pathway regulation. During plant growth and development, as well as under biotic and abiotic stress conditions, the expression/activity of different PAL gene/protein is, in fact, highly modulated and controlled by internal and external signals in stringent correlation with fluctuations in phenolic concentrations. Tomato contains a surprisingly large family of PAL genes (∼26 copies/diploid genome) widely dispersed in the genome. Nevertheless, differently to watermelon and other plants, in which many of the PAL gene loci are expressed in response to alternative stimuli, only a single sequence is expressed (Chang et al., 2008). In watermelon, of the 12 *PAL* genes identified, 6 are moderately or strongly expressed in the fruits, particularly in the pulp, suggesting their potential regulative roles in the *de novo* synthesis of phenolics and other secondary metabolites (Dong and Shang, 2013).

Redundant but distinctive *cis*-regulatory structures for stress responsiveness have been identified in watermelon and tomato PAL genes through promoter motif analysis. Feedback effects or post-translational modifications are also involved in PAL regulation. Furthermore the differential subcellular distributions of PAL isoforms and the formation of metabolons with C4H, facilitating metabolite channeling to *p*-coumaric acid, might provide an extra and unsuspected level of metabolic regulation, allowing precursors partition into different branch phenylpropanoid pathways (Boudet, 2007).

More than sixteen cytochrome P450 monooxygenases, including C4H and C3’H, have been recognized as important regulators in phenolic metabolism by irreversibly channeling 4-coumaroyl-CoA flow into specific pathway branches, contributing thus to the diversity and flexibility of phenolic metabolism. In particular, putative *cis*-acting elements have been identified in the promoter region of C4H genes, further suggesting a coordinated transcriptional regulation of the expression of the enzyme, repeatedly identified as rate limiting (Boudet, 2007). Schijlen et al. (2004) pointed out the presence of a well-synchronized control of the biosynthetic genes at transcriptional level, mediated by various transcription factors, outlining the levels of each compound to attain within plant cells. These phenols and flavonoids regulatory genes are tissue- and stress-dependent and have been classified in two families; the first with sequence homology to proto-oncogene c-MYB protein, and the other similar to the basic-Helix–Loop–Helix (bHLH) protein encoded by the proto-oncogene c-MYC (Mol et al., 1998).

Conjugation and compartmentalization processes have also a role in determining the levels of phenolics in plants. Members of the multi-antimicrobial extrusion protein family in synergism with vacuolar sorting proteins, glutathione *S*-transferases and ERFs (ERF1 and ERF4) have been proposed to finely orchestrate phenolic sequestration within the vacuole in a tomato introgression line carrying a 32 cM single homozygous chromosome segment from *Solanum pennellii* characterized by increased levels of total phenolics (Di Matteo et al., 2013).

Future investigations are required to better elucidate the complex regulation of phenylpropanoid pathway.

## Aroma Volatiles in Tomato and Watermelon Fruits

### Chemical Features and Functions

Fruits synthesize, store and release various volatile molecules perceived by humans as aromas by interacting with specific receptors of the olfactory epithelium. Aromas constitute a huge heterogeneous class of chemicals carrying alcoholic, aldehydic, ketonic, acid, ester and ether functional groups inserted on saturated/unsaturated, straight/branched or cyclic structures (Schwab et al., 2008). Some (primary odorants) bind/activate a single receptor and define one odor individually; other (secondary odorants) interact with multiple receptors, leading to a more complex smell perception (Baldwin et al., 2000). Aroma is essential for fruit flavor, and along with sugars and acids, noticeably affects food consumer acceptance and choice; thus, it is gaining growing consideration as major quality attribute of fresh and processed fruit products (Wang et al., 2016).

Tomato and watermelon fruit volatile profiles are extremely complex. In tomato, more than 400 molecules have been identified. However, only few (∼30) are assumed to effectively contribute the typical aroma of the fresh fruits and processed products based on concentration (>1 ppb), perception threshold (the level at which a compound can be detected by smell) and positive Log odor units (the logarithm of the ratio of the concentration of a component in a food to its perception threshold). According to Petro-Turza (1987) and Buttery et al. (1998), *(Z)*-3-hexenal, β-ionone, hexanal, β-damascenone, 1-penten-3-one, 3-methylbutanal, *(E)*-2-hexenal, *(Z)*-3-hexenol, 6-methyl-5-hepten-2-one, methyl salicylate and 2-isobutylthiazole are the major contributors driving the aroma of fresh, ripe tomato, though others may embellish the overall flavor as background notes. In watermelon Nijssen (1996) documented the presence of 75 different chemicals: 23 alcohols, 21 aldehydes, 12 furans, 8 ketones, 7 hydrocarbons, 2 lactones, 1 acid, and 1 oxide. However, those mostly contributing to the pleasant aroma notes of the ripe fruits include C6 and C9 aldehydes [*(Z)*-3-nonenal, *(Z,Z)*-3,6-nonadienal, *(E,Z)*-2,6-nonadienal, hexanal, nonanal, *(E)*-2-nonenal, *(E)*-6-nonenal], ketones (6-methyl-5-hepten-2-one, geranyl acetone), alcohols [*(Z)*-3-nonenol, *(Z,Z)*-2,6-nonadienol, hexanol, nonanol, *(Z)*-6-nonenol, *(E*,*Z)*-2,6-nonadienol] and their esters (Fredes et al., 2016). Watermelon fruits may also develop off-flavors such as *(E)*-6-nonenol responsible for the pumpkin-like fragrance (Kyriacou and Rouphael, 2018).

### Biosynthesis, Fruit Profiles and Regulation

Despite the relatively low rate of DNA sequence diversity within the cultivated germplasm, significant variation in the amounts and profiles of volatiles among genotypes and fruit fractions has been reported for both tomato and watermelon. A surprisingly large chemical diversity in volatile emissions (up to 3,000-fold change, expressed as ng/g fw/h) across 152 heirloom tomato cultivars was observed by Tieman et al. (2012). Regardless of cultivars, peels and pulp seem the main fractions influencing tomato fruit aroma, followed by the locular fluid and, barely, seeds (Buttery et al., 1998; Wang et al., 2016). Similarly, Lewinsohn et al. (2005a) found different aroma profiles in two red-fleshed watermelon cultivars, with Moon and Star showing significantly higher monoterpenes and norisoprenes than Crimson Sweet, and 6-methyl-5-hepten-2-one as predominant volatile molecule (157 ng/g fw). Geranial [*(E)*-citral] was, instead, the main aroma detected in Crimson Sweet (16 ng/g fw). Beaulieu and Lea (2006) identified 59 volatile compounds (12 for the first time) in five different fully ripe triploid seedless red watermelon cultivars. The authors confirmed that the characteristic aroma of freshly cut fruits is associated mainly with alcohols, aldehydes and ketones, accounting up to 81.6% of the total volatile compounds, and highlighted some differences between conventional seeded and seedless cultivars. No acid and ester volatiles were detected, in fact, in any of the seedless watermelon cultivars assayed, possibly related to the minimal production of ET, typical of these genotypes, required to initiate the cascade of enzymatic reactions leading to the synthesis of these aromas. Among seedless cultivars, Pure Heart was the only synthesizing hexanal as major aroma volatile, confirming the importance of genotype as major determinant of aroma composition (Beaulieu and Lea, 2006).

**FIGURE 6:**
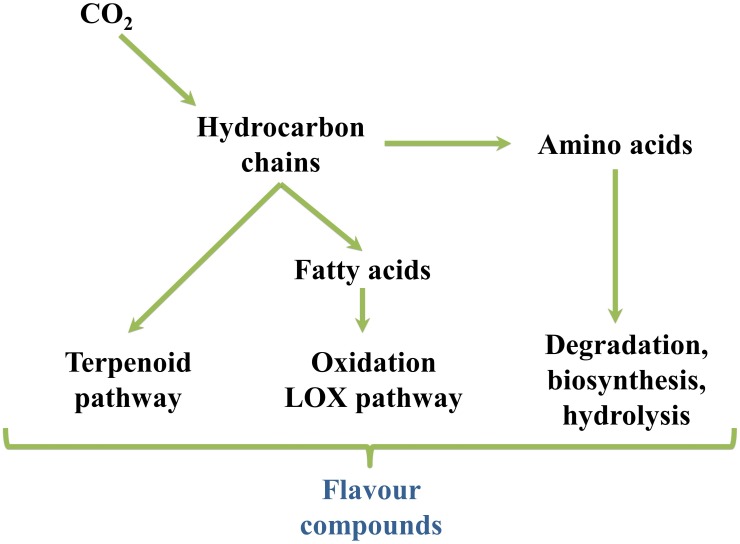
Overview of flavor compounds biosynthetic pathways.

The production of volatile aromas in tomato and watermelon, as well as in most other fresh fruits and vegetables, occurs through different metabolic pathways, but primarily via the catabolism of fatty acids, amino acids, glycosides, carotenoids and terpenoids (Figure 6) (El Hadi et al., 2013). Various enzymatic modifications (hydroxylation, acetylation, methylation, etc.) further increase the chemical complexity of the synthesized compounds. Besides, some volatiles, absent in the intact fruit, are produced as a result of tissue disruption occurring when the fruits are cut, squeezed or homogenized (Buttery et al., 1998).

Although the use of fast-evolving analytical tools is deeply affecting our knowledge on aroma biosynthesis, many steps have not been elucidated, yet. However, most are thought to be identical in different crops (El Hadi et al., 2013). Free fatty acids, particularly polyunsaturated linoleic (C18:2) and linolenic (C18:3), are the main precursors of most fruit aroma volatiles via *α*- and *β*-oxidation in intact fruits, or the LOX pathway in fresh-cut fruits and juices (Table 1) (Schwab et al., 2008). Two different LOX (9-LOX and 13-LOX), together with hydroperoxide lyases (HPLs) and a hydroperoxy cleavage enzyme, convert linoleic and linolenic acids to hexanal and *(Z)*-3-hexenal, respectively, through 9- and 13-hydroperoxy-intermediates. Hexanal and *(Z)*-3-hexenal are reduced to hexanol and *(Z)*-3-hexenol, respectively, by the reductase enzyme alcohol dehydrogenase (ADH). Further isomerization of *(Z)*-3-hexenal to *(E)*-2-hexenal can occur either enzymatically or non-enzymatically (Baldwin et al., 2000). In tomato, five LOX genes (Tomlox A, B, C, D, and E) are expressed during fruit ripening. Their suppression severely alters aroma and flavor synthesis suggesting them as a target for genetic modification (Pech et al., 2008). Furthermore, antisense targeting of phospholipase D (PLD-α), a key enzyme that initiates membrane deterioration and leads to the loss of compartmentalization and homeostasis during fruit ripening and senescence, yielded important quantities of the LOX-derived aldehydes *(E)*-pentenal and 2-hexenal after tomato tissue disruption probably due to the accumulation of high substrate levels of the LOX pathway indicating the existence of very intricate regulatory mechanisms (Oke et al., 2003).

**Table 1:** Overview of carotenoids-, fatty acids- and amino acids-derived aroma and flavor compounds biosynthetic pathways. LOX, lipoxygenase.

		Additional
Compounds	Reactions	information	Final aromatic products
**Carotenoids**			
Phytoene and phytofluene	Cleavage catalyzed by a family of carotenoid cleavage dioxygenases (CCDs)	Acyclic volatiles	Farnesylacetone Dihydroapofarnesal Geranylacetone 6-Methylhept-5-en-3-one
Lycopene, prolycopene, neurosporene and δ-carotene			Neral Geranial 6-Methyl-5-hepten-2-one 2,6-Dimethylhept-5-1-al 2,3-Epoxygeranial (*E,E*)-Pseudoionone
β-carotene		Cyclic volatiles	β-Ionone β-Cyclocitral Dihydroactinidolide
δ-carotene			α-Ionone
**Fatty acids**	β-Oxidation	Aroma volatiles in intact fruits	C_1_ to C_20_ straight-chain alcohols, aldehydes, ketones, acids, esters and lactones
	LOX	Aroma volatiles in fresh-cut fruits	
**Amino acids** (alanine, valine, leucine, isoleucine, phenylalanine, and aspartic acid)	Deamination/transamination, decarboxylation, oxide-reduction, and esterification		Alcohols, carbonyls, aldehydes, acids and esters


Two isoforms of ADH have been identified in tomato fruits: *LeADH2* has been found to be particularly active during ripening for the synthesis of *(Z)*-3-hexenol.

Several amino acids (alanine, valine, leucine, isoleucine, and phenylalanine) are involved in the production of aroma volatiles through complex reactions of deamination/transamination, decarboxylation, oxide-reduction and esterification (Table 1) (Schwab et al., 2008). This occurs typically in the fruit during ripening rather than upon cell disruption. Decarboxylases and deaminases transform the amino acids in aldehydes, whose low and high levels have been strictly correlated to pleasant and unpleasant fragrances, respectively (Tadmor et al., 2005). The antisense down-regulation of the decarboxylase gene in tomato yield a limited synthesis of phenylacetaldehyde and phenylethanol, while its overexpression increases phenylethanol, phenylacetaldehyde, phenylacetonitrile, and 1-nitro-2-phenylethane levels, significantly affecting tomato aroma (Tieman et al., 2006).

Glycoside hydrolysis also leads to the release of volatile aromas such as 3-methylbutyric acid and β-damascenone, phenylacetaldehyde, 2-phenylethanol, linalool, linalool oxides, hotrienol, α-terpineol, 4-vinylguaiacol, 4-vinylphenol, furaneol (Buttery et al., 1998).

Carotenoids are precursors of some important C8, C13, C18 linear and cyclic isoprene flavor volatiles through a pathway involving three steps: an initial oxidative cleavage by CCDs to apocarotenoids, an enzymatic transformation in polar aroma precursors, and finally the acid-catalyzed conversion to volatiles. The synthesis of carotenoid-derived volatiles occurs late during fruit ripening and is a good indicator to monitor the process (Auldridge et al., 2006). In tomato, two similar genes (*LeCCD1A* and *LeCCD1B*) were reported to encode for CCDs able to cleave multiple linear and cyclic carotenoids preferably at the 9,10 (9′,10′) locations leading to the synthesis of β-ionone and geranylacetone, major contributors of fresh tomato aroma, representing, therefore potential targets to genetically modify fruit aroma synthesis (Simkin et al., 2004).

Finally, some volatiles (e.g., α-copaene, linalool, neral, geranial, methyl salicylate, eugenol, benzaldehyde, and guaiacol) are formed from terpenoids (C10 and C15), related to lignin or of unknown origin, but most do not seem to contribute to the aroma of either tomato or watermelon fruits.

Volatile and aroma profiles of fruits are continuously changing as several endogenous and exogenous factors/stimuli affect their synthesis and accumulation, generally, the same responsible of fluctuations in their originating precursors, including ripening stage, growing system and conditions and pre- and post-harvest practices (Baldwin et al., 2000). Although their importance and potential practical application in controlling a chief fruit quality attribute, there is a lack of knowledge about the cross-talk of the different factors in determining the potential of aromas in tomato and watermelon (El Hadi et al., 2013).

During tomato and watermelon ripening a series of qualitative and quantitative biochemical changes occur and affect the volatile profile of fruits. Several authors reported a general increase in aroma concentration along with both natural and artificial ripening, especially through the breaker to red stage transition, however, the expression patterns and concentrations of the individual compounds follows a complex and largely genotype-dependent pattern of change, with some remaining stationary or even decreasing (Renard et al., 2013). It is well known that early harvest of tomatoes before full ripeness impairs volatile production, resulting in poor flavor quality fruits. Fruits harvested 1-day before the breaker stage revealed significantly higher levels of 1-penten-3-one, *(Z)*-3-hexenal, 6-methyl-5-hepten-2-one, 2-isobutylthiazole and geranylacetone than those harvested 5-day before the breaker stage (Maul et al., 1998). Baldwin et al. (1991) assessed aroma volatiles in two tomato cultivars (Sunny and Solar Set) at five ripening stages. Hexenal, hexenal acetone, 6-methyl-5-hepten-2-one, geranylacetone, *(Z)*-3-hexenol, acetaldehyde, *(Z)*-3-hexenal, *(E)*-2- and 2-isobutylthiazole peaked at the turning, pink, or red-ripe stages. Eugenol concentrations decreased during ripening regardless the cultivar and 1-penten-3-one decreased only in the cultivar Sunny. Ethanol and *(E,E)*-2, 4-decadienal concentrations remained unchanged or little fluctuated during ripening. Except for ethanol and hexanal in mature tomato fruits, Solar Set tomato fruits showed higher flavor content than Sunny. Accordingly, the activity of the tomato microsomal LOX increased between the green and turning stage and decreased as the fruit reached full ripeness.

It has also been reported that aroma volatiles differ between vine and off-vine ripened tomato fruits. Benzaldehyde, citronellyl propionate, citronellyl butyrate, decanal, dodecanal, geranyl acetate, geranyl butanoate, nonanal, and neral are synthesized and emitted at higher levels in vine-ripened tomato fruits than in off-vine ripened fruits, which in turn release increased levels of other compounds including butanol, 2,3-butanedione, isopentanal, isopentyl acetate, 2-methyl-3-hexanol, 3-pentanol and propyl acetate (Madhavi and Salunkhe, 1998; Ilahy et al., 2018). The development of off-flavors is associated with the increased productions of 2-methyl-1-butanal, mostly in artificially ripened tomato fruits.

**FIGURE 7:**
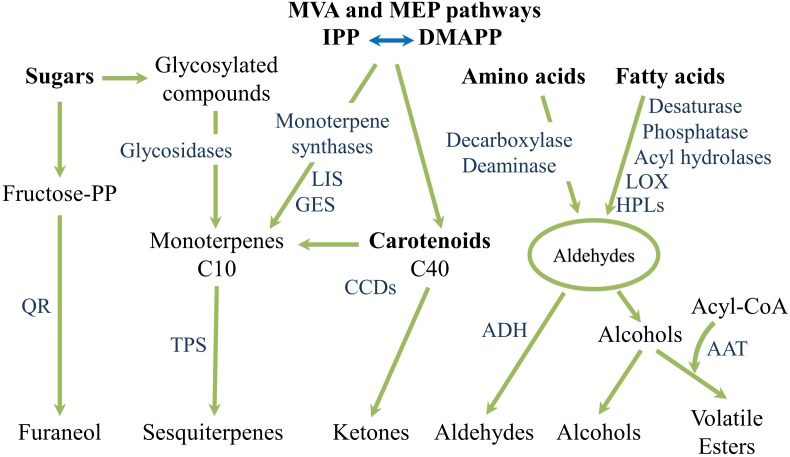
Overview of the fatty acids-, amino acids- and carotenoids-derived aroma and flavor compounds biosynthetic pathways. AAT, alcohol acyl-transferases; ADH, alcohol dehydrogenase; CCDs, carotenoid cleavage dioxygenases; DMAP, dimethylallyl diphosphate; GES, geraniol synthase; HPLs, hydroperoxide lyases; IPP, isopentenyl pyrophosphate; LIS, S-linalool synthase; LOX, lipoxygenase; QR, quinone oxidoreductase; TPS, terpene synthase.

It is worthwhile to mention that since key aroma volatiles in watermelon and tomato derive from carotenoid catabolism, differences in carotenoid profile underlie differences in their composition (Lewinsohn et al., 2005a; Liu et al., 2012; Kyriacou and Rouphael, 2018). Lewinsohn et al. (2005a) comparatively evaluated the volatile profiles of tomato and watermelon genotypes bearing different carotenoid biosynthetic gene mutations (*r*, *t*, *Del*, and *B*) revealing a strong overlap in the two crops for the same fruit flesh colouration and proposing that color differences induce similar profile changes in both tomato and watermelon fruits. Thus, ripe fruits of the red tomato and watermelon cultivars share the same major norisoprenoid volatiles 6-methyl-5-hepten-2-one, farnesyl acetone, pseudoionone and geranial; the yellow ones have farnesyl acetone as main isoprenoid volatile. High concentrations of farnesyl acetone, geranyl acetone and 6-methyl-5-hepten-2-one characterize orange *tg* tomato mutant and its watermelon equivalent Orangelo. In the orange *B* tomato and watermelon genotypes the main volatiles were, in descending order, dihydroactinidolide, farnesyl acetone, 6-methyl-5-hepten-2-one, β-ionone, β-cyclocitral, geranial, 2,3-epoxygeranial, neral, pseudoionone and dihydro-apo-*(E)*-farnesal. In the *Del* mutants, the main volatiles were 6-methyl-5-hepten-2-one, farnesyl acetone, α-ionone and geranial. Cyclic aroma compounds such as β-ionone, β-cyclocitral and dihydroactinidolide are the result of β-carotene cleavage, α-ionone derive, instead, from δ-carotene. Acyclic aroma volatiles are produced from the oxidative cleavage of phytoene, phytofluene, lycopene, pro-lycopene, and neurosporene (Table 1 and Figure 7).

Field grown tomatoes have higher levels of aroma volatiles than greenhouse-grown fruits, while other pre-harvest factors such as fertilization and watering all influence volatile composition depending on the levels.

Postharvest handling have a deep impact on the aroma compounds of fresh tomato and watermelon fruits. Tomato fruits mild chilling and refrigeration lead to significant changes in the levels of 3-methylbutanal, linalool, guaiacol, hexanol, *(E)*-2-hexenal and *(E)*-3-hexenol, possibly due to a decrease of fruit ADH activity (Sanchez et al., 2009; El Hadi et al., 2013). Controlled atmosphere reduces the emitted volatiles from fresh product following the inhibition of ET production (Mattheis et al., 2005). In addition, the application of methyl jasmonate was reported to alter the biosynthesis of different aroma components and minimize aroma post-harvest depletion in climacteric and non-climacteric fruits (De la Peña et al., 2010; El Hadi et al., 2013).

The composition of aroma volatiles in tomato and watermelon fruits differs between intact fruit, sliced fruits and homogenates. After tomato tissue disruption, some compounds appear [e.g., *(E)*-2-pentanal and geranial], other increase [e.g., *(Z)*-3-hexenal, *(E)*-2-hexenal, hexanal, *(E)*-2-heptenal, 1-penten-3-one, 1-penten-3-ol, geranylacetone, 2-isobutylthiazole, 1-nitro-2-phenylethane] and other remain unchanged [3-methylbutanol, pentanol, *(Z)*-3-hexenol, hexanol, 6-methyl-5-hepten-2-one, phenylacetaldehyde and 2-phenylethanol] (Baldwin et al., 2000).

Studying the quality characteristics of watermelon slices of the seedless cultivar Sugar Heart stored under modified atmosphere conditions at 5°C, Saftner et al. (2006) found a general decrease in the concentration of most volatiles, except *(Z)*-6-nonen-1-ol, 6-methyl-5-hepten-2-one, 6-methyl-5-hepten-2-ol, *(Z)*-6-nonenal, *(E)*-2-nonenal, nonan-1-ol, β-cyclocitral and geranyl acetone whose levels remained unchanged or transiently increased. The accumulation of *(Z)*-6-nonen-1-ol is of particular importance as it has been associated with the pumpkin-like off odor. The authors also reported that whole fruit pre-treatments with 1-methylcyclopropene could prevent ET-induced adulterations of watermelon slices volatile aromas, but not the development of off-odors.

The pH, also, has been demonstrated to play a fundamental role on the stability of fresh watermelon juice aromas; acid values in the range 3.0–5.6 promote the development of off-flavors, while the juice is more stable at pH between 6.0 and 7.0 (Yajima et al., 1985).

Acting to promote the transcription and translation of numerous ripening-related genes, ET has an important role in aroma volatile development in both tomato and watermelon fruits (Wyllie et al., 1998). ET is known as lipoxygenase activator, promoting the synthesis of the fatty acid derived volatiles as reported for tomato and climacteric and non-climacteric melon cultivars (Obando-Ulloa et al., 2008). ET also affect amino acid and carotenoid derived volatiles by controlling the synthesis of their direct precursor, in fact the synthesis of phenylalanine, leucine, and isoleucine, as well as some steps of the carotenoid metabolic pathway, are strongly under ethylene control during ripening (Klee and Giovannoni, 2011).

## Vitamin C in Tomato and Watermelon Fruits

### Chemical Features, Distribution and Functions

Ascorbic acid (AsA), also known as Vitamin C, is a multifunctional metabolite common in all photosynthetic eukaryotes and essential for plant growth and development. Besides its role in photosynthesis as enzyme cofactor, AsA is a major hydrophilic antioxidant able to efficiently scavenge free radicals and reduce high oxidation states of iron to Fe^2+^. In fact, it is easily oxidized with loss of one electron to monodehydroascorbic acid (MDHA) and then, losing a second electron, to dehydroascorbic acid (DHA) (Smirnoff, 1996). In addition, AsA and DHA can act as signaling agents mediating the response to biotic and abiotic effectors, including pathogens, ozone, oxidizing agents, and water loss (Mellidou et al., 2012).

Ascorbic acid occurs in all plant cell organelles/compartments at intracellular concentrations of 2–25 mM and appears differentially distributed depending on subcellular localization and tissue. In *Arabidopsis* and *Nicotiana* mesophyll cells, the highest AsA levels were found in the cytosol, followed by peroxisomes, nuclei, mitochondria, chloroplasts and vacuoles (Smirnoff, 1996). It has also been detected in the apoplast (along with an extracellular isoform of ascorbate oxidase), where represents the only significant redox buffer and appears involved in the non-enzymatic scission of plant cell wall polysaccharides by triggering the *in situ* production of hydroxyl radicals via the Fenton reaction. This phenomenon is particularly important during fleshy fruit ripening and has been proposed to play a role in tomato softening (Dumville and Fry, 2003; Zechmann, 2011). DHA is also detectable in plant tissues, but its role is controversial as some authors assume it comes from the artifactual oxidation of AsA occurring during sample processing. However, the presence of AsA and DHA uptake/efflux high affinity carriers on plant cell membranes argues in favor of defined physiological functions. Besides, the uptake of AsA by chloroplasts is controlled by DHA concentrations (Smirnoff, 2018).

In humans, AsA is an essential micronutrient required for normal metabolic functioning of the body, especially for collagen, carnitine and neurotransmitters biosynthesis. Many health benefits have been attributed to ascorbic acid such as antioxidant, anti-atherogenic, anti-carcinogenic, immunomodulatory, etc. However, lately the health benefits of ascorbic acid have been the subject of debate and controversies. Humans and other primates have lost the ability to synthesize vitamin C, thus, it must be introduced with the diet. The Recommended daily intake (RDI) for ascorbic acid for non-smoking adults is set to be 75 and 90 mg/day for females and males, respectively (Monsen, 1996).

### Fruit Concentration, Distribution, and Regulation

Tomato and watermelon fruits are good sources of vitamin C accumulating, on average, 12.6 and 8.1 mg/100 g fw as total AsA, respectively^2^ though, in both crops, genotype, ripening stage, growing season and pre/post-harvesting practices have been reported to strongly affect its levels (Isabelle et al., 2010; Ilahy et al., 2011, 2016b, 2018; Tlili et al., 2011a,b). In the ordinary tomato cultivar Rio Grande, for instance, a significant variation of AsA and DHA concentrations in the range 67.8–108.0 mg/kg fw and 73.4–152.0 mg/kg fw was reported during ripening (Ilahy et al., 2011), while HLY tomato cultivars showed broader intervals (55.8–180 mg/kg fw and 37.8–213 mg/kg fw for AsA and DHA, respectively). The dynamic of change of total vitamin C (AsA + DHA) was also genotype-dependent, with some cultivars (Rio Grande, HLY18, and HLY13) peaking at the orange-red ripening stage, while Lyco2 showing two peaks at the green–orange and red-ripe stages (Siddiqui et al., 2018). Differences in the total vitamin C and AsA accumulation profiles during ripening were also reported between the “low-AsA” tomato cultivar Ailsa Craig and the “high-AsA” cultivar Santorini, although both displayed a characteristic peak at the breaker stage (Mellidou et al., 2012).

Al-Harbi et al. (2017) suggested that grafting is ineffective in improving vitamin C content in tomato fruits. A 14–20% decrease was, instead, reported by Di Gioia et al. (2010) in open field grown fruits of the heirloom “Cuore di Bue” cultivar grafted onto two interspecific (*S. lycopersicum* ×*Solanum habrochaites*) rootstocks (Beaufort F1 and Maxifort F1). Mycorrhiza treatment applied at sowing and planting during two consecutive seasons determined an increase of vitamin C content under dry conditions and a decrease under wet conditions (Nemeskéri et al., 2019). Helyes et al. (2019) focused on the simultaneous effect of three water supply regimes and treatments with different concentrations of the bio-fertilizer Phylazonit on the physiology and quality of the processing tomato cultivar Uno Rosso. The authors revealed highly significant effects of water supply regimes and bio-fertilization on the content of AsA that increased from 221.8 to 369.1 μg/g fw.

Recently, Ilahy et al. (2019) suggested that tomato cultivars carrying *hp* mutations could be used in organic farming to overcome the general observed antioxidant content and functional quality decline in traditional tomato cultivars. It has been reported that storage influence tomato health-promoting properties negatively or positively according to storage duration and conditions. Ascorbic acid content was found to decrease during storage and the greater the levels of different bio-actives initially accumulated in the fruits, the lower is the effect of the storage on ascorbic acid content (Siddiqui et al., 2018).

Similar to tomato, total vitamin C exhibited a cultivar-dependent dynamic of change during watermelon fruit ripening. The highest amount of total vitamin C was recorded at the red-ripe and pink stages in Crimson sweet and P503 cultivars, respectively, but at the white stage in Giza, Dumara and P403 cultivars. In the flesh of red-ripe fruits, total vitamin C values ranged from 119.7 to 204.0 mg/kg fw in the cultivars P403 and Giza, respectively. Vitamin C levels also varied in relation to the growing location, in fact, Giza, Dumara and P403, exhibited lower total vitamin C content when grown in Southern Italy than in Northern Tunisia (Tlili et al., 2011a,b).

Choo and Sin (2012) assayed the AsA content in the flesh of different flesh-colored watermelons. Yellow cultivars were found to have significantly lower AsA levels (52.1 mg/kg fw) than red ones (86.3 mg/kg fw). Otherwise, Isabelle et al. (2010) found higher ascorbic acid content in yellow than in red watermelon ripe fruits. These differences have been attributed to the strong influence by genotype, environmental conditions, harvest and post-harvest practices on AsA anabolic, catabolic and recycling pathways. It has also been reported that AsA content of the watermelon rind and seed were significantly lower compared with the pulp (Johnson et al., 2013).

Tlili et al. (2011b) reported significant variations in total vitamin C content between watermelon cultivars and sampling areas within each cultivar with values ranging from 105 to 240 mg/kg fw in Aramis and Dumara, respectively. Besides, a genotype-dependent effect of sampling area on the total vitamin C content was noticed with the highest values recorded for peripheral and stem-end areas and the lowest obtained for heart and blossom-end areas.

Although data of the effect of growing season and pre/post-harvesting practices on total vitamin C in watermelon cultivars are scarce, Leskovar et al. (2004) reported that vitamin C and lycopene are not affected by water stress in watermelon fruit. Proietti et al. (2008) demonstrated that dehydroascorbate and vitamin C contents were increased, respectively, by 13 and 7% in grafted compared to ungrafted plants.

Hdider et al. (2019) recently reviewing the available data regarding the nutritional composition, antioxidant properties and health benefits of watermelon fruits, reported that temperature and postharvest storage conditions and duration are the most important factors to extend shelf-life and conserve quality of fresh watermelon including vitamin C content.

**FIGURE 8:**
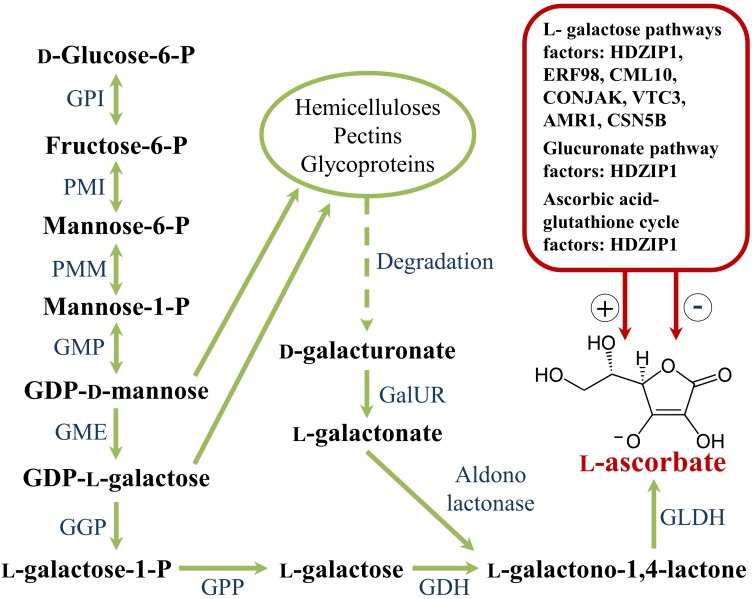
Key biosynthetic pathways of ascorbic acid in plants. GalUR, D-galacturonic acid reductase; GDH, L-galactose dehydrogenase; GGP, GDP-L-galactose phosphorylase; GLDH, L-galactonolactone dehydrogenase; GME, GDP-D-mannose-3,5-epimerase; GMP, GDP-D-mannose pyrophosphorylase; GPI, glucose-6-phosphate isomerase; GPP, L-galactose-1-P phosphatase; PMI, phosphomannose isomerase; PMM, phosphomanno mutase.

Even if alternative routes involving uronic acids, L-gulose and myo-inositol may contribute to enhance AsA levels in higher plants, D-mannose/L-galactose (Smirnoff-Wheeler) pathway (Figure 8) represents the primary biosynthetic route in most crops, including tomato (Wheeler et al., 2015). The pathway appears regulated at both transcriptional and post-transcriptional levels; however, the knowledge on the mechanisms that control AsA synthesis and metabolism is still limited. Based on metabolite analyses, non-labeled and radiolabelled substrate feeding experiments, enzyme activity measurements and gene expression studies conducted on different crop species, several key regulatory components of the pathway have been proposed. These include GDP-D-mannose pyrophosphorylase (GMP), GDP-D-mannose-3,5-epimerase (GME), GDP-L-galactose phosphorylase (GGP), L-galactose-1-P phosphatase (GPP), L-galactose dehydrogenase (GDH) and L-galactonolactone dehydrogenase (GLDH). Phosphomannose isomerase (PMI) and phosphomanno mutase (PMM), the enzymes catalyzing the early steps of the pathway, instead, do not seem determinant in modulating AsA level (Ioannidi et al., 2009; Mellidou et al., 2012; Mellidou and Kanellis, 2017; Tao et al., 2018).

Various regulatory proteins and transcription factors have been proposed to positively or negatively affect the content of AsA. For instance, HD-ZIP1 increases the transcription of GME, GPP, GGP, and GLDH; ERF98 that of GMP, GME, GGP, GLDH and GDH, CML10, and CONJAK1/2 stimulate PMM and GMP activity, respectively, and VTC3 influences positively GGP posttranslational modification. AMR1 and CSN5B have been proposed to negatively affect the post-translational modulation of GMP and the transcription of all intermediate enzymes of the D-mannose/L-galactose pathway (Mellidou and Kanellis, 2017).

In tomato, GPP appears as the main regulatory enzyme of the D-mannose/L-galactose pathway. Its expression is ripening and ethylene regulated, and affected by light and stress conditions (Ioannidi et al., 2009). GPP is also subjected to translational regulation through a *cis*-acting upstream open reading frame in the 5′-UTR that represses GGP translation under high AsA concentration allowing a rapid and feedback responsive control of its biosynthesis under demanding conditions (e.g., high light, low temperatures) (Bulley et al., 2012; Mellidou et al., 2012; Mellidou and Kanellis, 2017).

The regulatory role of the other enzymes of the pathway is still controversial in tomato fruits. For example, GLDH gene expression followed an opposite profile when compared with the corresponding melon gene, which accumulated during ripening, coinciding with elevated levels of AsA (Pateraki et al., 2004), indicating different means of regulating AA synthesis in these two fruit.

In red-ripe tomato fruits, similarly to other crop species including strawberry, apple, orange, and grape, a less important alternative pathway operates in maintenance of AsA pools. This pathway uses galacturonate as a substrate of aldono lactonase (AL) to give L-galactono-1,4-lactone that is subsequently converted to AsA by GLDH. Galacturonate is a pivotal compound with double importance since it is also the elementary unit forming cell wall pectins and as AsA primer (Mellidou and Kanellis, 2017).

The ascorbate pool undergoes turnover in plants. The final stable products of AsA catabolism differ between species and in tomato are represented by oxalate, threonate and oxalyl threonate (Smirnoff, 2018). The rate of degradation and/or the capacity for AsA regeneration are also responsible for the control of AsA levels in tomato fruits. After oxidation to MDHA and DHA AsA is regenerated by the ascorbate-glutathione cycle and, particularly, the intervention of glutathione, DHA and MDHA reductases (GSHR, DHAR, and MDHAR, respectively) (Foyer and Noctor, 2011; Mellidou and Kanellis, 2017). Within this cycle, the HD-ZIP1 transcription factor has been reported to influence positively the transcription of both key enzymes DHAR and MDHAR. It also enhance the transcription of oxygenase (MIOX), enzyme involved in the synthesis of D-glucuronic acid from myo-inositol. These mechanisms of regulation have been proposed to be cultivar-dependent. In fact, in the low-AsA cultivar Ailsa Craig, this alternative route of AsA biosynthesis may supplement biosynthesis via L-galactose, while in the high-AsA cultivar Santorini, enhanced AsA recycling activities appear to be responsible for AsA accumulation in the later stages of ripening.

At the best of our knowledge, there are no specific reports on AsA regulation in watermelon fruits. Nevertheless, foliar application of gibberellic acid was reported to increase the AsA content in other Cucurbitaceae as also reported for tomato fruits (Basu et al., 1999).

## Harmful Effect of Excess Bioactive Compounds on Human Health

Consuming plants for their presumed health benefits has occurred since early civilizations. Phytochemicals are generally thought to be safe for consumption because they are produced naturally. However, this is not always the case as many natural compounds found in several commonly consumed plants are potential deleterious for health, especially if assumed in excessive amount. Most plant bioactive compounds are plant secondary metabolites and some exert potent antioxidant activities and/or disease-combatting abilities when consumed within specific doses (Bruno et al., 2018; Siddiqui et al., 2018).

Based on the rising interest of costumers for natural and isolated bioactive compounds of plant origin, and the consequential risk of excessive intake, there is an urgent need to evaluate their real or potential functionality on human health and/or wellbeing and develop scientifically based recommendations for a safe intake.

Although the increasing evidence regarding the negative correlation between fruits and vegetables dietary consumption and the incidence of various chronic diseases, the observed effects are hardly attributable to a single compound or class of compounds (Erdman et al., 2009; Russo et al., 2017; Durante et al., 2019). Recently, various researchers reported a double-edged sword function (antioxidant/pro-oxidant activity) for many phytochemicals, including lycopene, phenols and flavonoids (Skibola and Smith, 2000; Tokaç et al., 2013; Bacanli et al., 2017) depending on various factors (Fernando et al., 2019). Generally, high concentrations, low pH and/or the presence of redox-active transition metal ions, causes certain phytochemicals to exhibit pro-oxidant activity (Eghbaliferiz and Iranshahi, 2016).

Clinical trials using different isolated molecules, including β-carotene, vitamin E and vitamin C, resulted disappointing (Bjelakovic et al., 2008; Erdman et al., 2009). Hennekens et al. (1996) reported that long-term supplementation with β-carotene has no beneficial effect on the incidence of malignant neoplasms and cardiovascular diseases. Unlike essential nutrients, bioactive compounds act through various and complex mechanisms to confer, often, a similar outcome. The body defense system is poly-sided, consequently the treatment with an isolated molecule will probably arise little effect on the incidence of chronic disease except in the case of early deficiency (Erdman et al., 2009).

Additionally, it has been reported that bioactives acts in synergy. According to Erdman et al. (2009), lycopene may synergize with other antioxidant compounds leading to the supposed and/or observed protective effect in some of the cardiovascular or cancer protection and hence it might not be the only important phytochemical in tomato and watermelon fruits. Besides, the importance of the catabolic products of lycopene (lycopenoids) on health is emerging due to growing evidences on their direct effect on gene expression and modulation (Erdman et al., 2009). It has been demonstrated that at concentrations ranging from 0.5 to 8 μM, lycopene does not induce DNA impairment, however, above 12 μM, it seems to prompt DNA damage in human cells (Tokaç et al., 2013; Bacanli et al., 2017).

Many flavonoids mimic mutagenic agents when ingested at high/cytotoxic levels. Quercetin was demonstrated to induce frame-shift mutation and DNA pair-base substitutions as well to prompt chromosome abnormalities and exert pro-oxidant activity (Skibola and Smith, 2000). Arif et al. (2015) focused on the cellular DNA damage initiated by different flavonoids and concluded that myricetin exhibited the highest level of DNA degradation followed by fisetin, quercetin, kaempferol, and galangin. Scheffler et al. (2018) reported that catabolism of AsA and DHA generates highly reactive carbonyl-intermediates able to induce glycation of cell proteins, an irreversible and relatively slow process thought to underlie the functioning of the biological clock that controls aging and involved in the onset of several age-related and degenerative diseases. Recently, Parent et al. (2018) reported that vitamin C intake is ineffective in prostate cancer prevention.

Therefore, it is important to focus accurately on dose-response relationship between ingested single/group of antioxidant and reactive oxygen species balance in human body in order to develop scientifically based recommendations for a safe intake. Future intervention studies had better to emphasize, the cumulative, synergistic and antagonistic effect of the entire bioactive pool present in fruits and vegetables rather than simply concentrating on the effect of single phytochemical compound without sufficient consideration to the potential of associated compounds.

## Conclusion

Tomato and watermelon functionality resides deeply inside the presence of high levels of carotenoid pigments but it goes far beyond color as many other botanicals synergistically contribute to the hailed health-promoting properties of both fruits. The quantities and profiles of all these bioactive compounds, as well as their metabolic pathways and regulatory mechanisms, show similarities and differences between the two crops but, concurrently, appear substantially genotype-dependent despite the low level of genetic diversity in the cultivated germplasm.

A complex interweaving of anabolic, catabolic, and recycling reactions, finely regulated at multiple levels and with temporal and spatial precision, ensures a certain homeostasis in the concentrations of carotenoids, phenolics, aroma volatiles and AsA within the fruit tissues. Nevertheless, several exogenous factors including light and temperature conditions, pathogen attack, as well as pre- and post-harvest manipulations can drive their amounts far away from homeostasis. These adaptive responses allow crops to better cope with abiotic and biotic stresses but may severely affect the supposed functional quality of fruits.

Although the knowledge on the cross-talk between the plethora of stimuli and effectors involved in the regulation mechanisms is still limited, tomato and watermelon have been subjected to successful technological interventions aimed at fortifying and/or diversify the health-promoting attributes of the fruits either through metabolic engineering or the manipulation of pre- and post-harvest factors. However, based on the safety concerns recently raised, particularly about the seemingly indiscriminate addition of botanicals to foods, the real or potential functionality of these nutritionally fortified fruits must be supported by intervention studies on the cumulative, synergistic and antagonistic effect of their entire bioactive phytocomplex.

## Author Contributions

RI and ML conceptualized the idea of this review. RI, IT, ML, and MS scanned the literature, retrieved and processed manuscript referenced in the review, and wrote the manuscript. RI, ML, and CH critically reviewed the manuscript and enriched the key parts of the manuscript. All authors contributed to the revision of the manuscript prior to submission.

## Conflict of Interest Statement

The authors declare that the research was conducted in the absence of any commercial or financial relationships that could be construed as a potential conflict of interest.

## Footnotes

1 http://www.fao.org/faostat/en/#data/QC

2 https://ndb.nal.usda.gov/ndb
